# Spatio-temporal optical coherence imaging and tomography for *in vivo* applications

**DOI:** 10.1117/1.JBO.31.11.113504

**Published:** 2026-05-25

**Authors:** Maciej Wojtkowski

**Affiliations:** a International Centre for Translational Eye Research, Warsaw, Poland; b Polish Academy of Sciences, Institute of Physical Chemistry, Warsaw, Poland; c Nicolaus Copernicus University, Institute of Physics, Torun, Poland

**Keywords:** imaging *in vivo*, spatio-temporal optical coherence imaging, optical coherence tomography, full field imaging, Fourier domain OCT

## Abstract

**Significance:**

Strong scattering in biological tissues limits the achievable imaging depth and produces noiseboth coherent and incoherentthat impairs contrast in volumetric *in vivo* reconstructions. By dynamically tailoring spatial and temporal coherence to suppress scattered photons, spatio-temporal optical coherence imaging (STOC) enables high-contrast, high-resolution *in vivo* visualization, with direct applications in ophthalmic diagnostics and complex tissue imaging.

**Aim:**

The primary aim of this work is to formalize and validate the theoretical foundations and practical implementations of STOC and its three-dimensional volumetric extension, spatio-temporal optical coherence tomography (STOC-T). Specifically, the study seeks to demonstrate their ability to enhance imaging performance in scattering media, benchmark against current optical coherence methods, and assess their readiness for clinical application.

**Approach:**

This work mainly reviews advances in spatio-temporal optical coherence imaging and tomography, and includes an original description of filtering multiply scattered signals derived from a generalized diffraction model, showing that application of STOC-T obeys the first Born approximation.

**Results:**

Spatio-temporal optical coherence Imaging and tomography markedly improved image fidelity in highly scattering media, demonstrated by enhanced fringe contrast and reduced incoherent background across phantoms and biological tissues. STOC-T achieved high-resolution, high-contrast *in vivo* visualization of challenging structures, including retinal layers and choroidal microvasculature, with markedly improved delineation compared with conventional coherence imaging. Quantitatively, STOC-T extended effective imaging depth while preserving lateral resolution (∼5  μm) and exhibited coherence noise suppression and high phase stability. Performance metrics revealed contrast improvements in retinal ganglion cell soma imaging, with shot-noise-limited sensitivity attainable under appropriate conditions. Practical limitations such as high data volume, camera bandwidth demands, and computational load were identified, alongside potential pathways for further enhancement through optimized phase coding and adaptive acquisition strategies.

**Conclusions:**

Spatio-temporal optical coherence imaging and tomography enable high-contrast, deep volumetric imaging with improved resolution by combining phase modulation, spectral sweeping, and coherence control. Validated *in vivo*, it resolves fine retinal/choroidal structures beyond conventional OCT limitations. Its simple hardware and computational emphasis position it for clinical translation, despite challenges in data volume and acquisition demands.

## 1 Introduction

Achieving *in vivo* optical microscopy with quality comparable to that attained in fixed specimens remains a formidable challenge. The fundamental barrier is the complex structure of biological tissue, which induces intense light scattering. This scattering dramatically reduces the number of undisturbed (ballistic) photons that can penetrate to deeper layers and return to the detector, thereby degrading image contrast. There is no single universally accepted physical model of image formation in scattering media; rather, a hierarchy of approaches is employed, each valid within a specific regime. Wave-based formulations derived from Maxwell’s equations, such as the Born approximation or Rytov approximation,^1^^,^^2^ accurately describe weak scattering but fail in the multiple-scattering limit, whereas the radiative transfer equation and its diffusion approximation capture multiple scattering of intensity at the expense of phase and interference information essential for coherent imaging.^3^ Consequently, bridging these regimes remains an open problem, motivating hybrid strategies that integrate statistical optics with coherence theory.

In this article, a practical framework is introduced that combines statistical averaging with generalized diffraction theory. The image formation process in a scattering medium is formulated in an intuitive, algebraic manner using the cross-correlation matrix formalism referenced to an ideal mirror image model. Within this representation, spatiotemporal optical coherence (STOC) imaging is introduced as an implementation of this framework.

In this view, STOC is not a standalone physical model of light propagation, but a hybrid measurement paradigm that leverages coherent wave optics and controlled statistical averaging to suppress multiply scattered contributions. Rather than explicitly modeling complex scattering processes, it engineers the illumination and detection conditions to preferentially retain weakly scattered, phase-stable components.

### 1.1 Optical Cross-talk as a Mechanism of Image Degradation in Scattering Media

Spatial heterogeneity of the refractive index, on a scale comparable to, or slightly larger than, the imaging resolution, leads to delocalization of image points relative to their original positions. In deep-tissue imaging, where backscattered light from optically inhomogeneous media is detected, such delocalization typically manifests as optical crosstalk (OC) between the transverse propagation modes of light - transverse electro-magnetic modes (TEMs).^4^ A conventional remedy for OC in classical microscopy is physical axial sectioning, e.g., imaging thin tissue slices or single-cell layers. Alternatively, in thick samples where physical preparation of the specimen is impractical, optical axial sectioning (OAS) by means of confocal microscopy offers a viable solution.^5^ In confocal microscopy, a spatially coherent beam is focused into the sample, and a small aperture in front of the detector rejects out-of-focus light,^6^ so that scatterers located only within the tightly focused Gaussian beam, where the wavefront remains locally flat in cross-section, contribute to the detected signal. This is the so-called “confocal gate” (CG), which defines the effective thickness of the sampled layer.^7^ The CG both enables axial sectioning and reduces optical crosstalk. However, to maintain high axial resolution and effective OAS, confocal microscopes must operate at short working distances and rely on high spatial coherence of the illumination beam to maximize the power density. These constraints limit the applicability of confocal microscopy in strongly scattering, unprocessed biological tissues.

### 1.2 Partial Temporal Coherence in *In Vivo* Imaging

A major advance in *in vivo* imaging was achieved by enhancing optical axial sectioning (OAS) through exploitation of light’s temporal coherence. In this approach, implemented in interferometric systems such as Optical Coherence Tomography (OCT), the axial sectioning performance becomes effectively decoupled from the numerical aperture (NA) of the illumination optics.^8^ By combining conventional confocal gating with temporal-coherence gating (tCohG), through broadband (polychromatic) illumination and an interferometric detection scheme, the axial resolution (i.e., the thickness of the optically selected slice) is determined by the spectral bandwidth of the light source, rather than the NA of the imaging lens.^9^^,^^10^ In practice, this dual-gating strategy has improved OAS in *in vivo* imaging by roughly an order of magnitude relative to confocal microscopy alone.^11^ Importantly, tCohG also mitigates OC arising from scattering within tissue, thereby enhancing contrast in highly heterogeneous media.^7^^,^^12^^,^^13^ Nevertheless, this enhancement comes at a cost because balancing CG and tCohG often forces a trade-off between lateral resolution and imaging depth. As the CG usually defines the effective axial imaging range, achieving deep imaging while preserving lateral detail frequently requires compromise. As a result, volumetric reconstructions may suffer from anisotropic resolution, complicating accurate interpretation in three dimensions. One pragmatic response to this limitation has been the introduction of dynamic focusing; i.e., refocusing during acquisition to maintain high lateral resolution across the depth.^14^^,^^15^ However, this approach inevitably increases acquisition time, which is critical in live imaging contexts.

Lastly, as with all imaging modalities that employ spatially coherent illumination (including both confocal microscopy and OCT), the high spatial coherence needed for efficient light delivery and detection also gives rise to speckle, which together with OC degrades image contrast and resolution.^16^^,^^17^ Historically, efforts to improve image quality in confocal microscopy and OCT have focused on reducing speckle contrast through averaging or other postprocessing schemes.^18^^–^^23^ Yet even with these strategies, full diffraction-limited resolution in all dimensions remains elusive because averaging suppresses noise and artifacts at the expense of spatial detail, effectively reducing the process to a form of coarse spatial smoothing by spatial or angular compounding.

Recent studies have also explored alternative strategies for mitigating multiple scattering and enhancing depth sensitivity in OCT, including spatially offset detection schemes. In particular, it has been demonstrated that spatially offset OCT can provide improved sensitivity to multiply scattered light and deeper tissue structures, supported by detailed forward models of light propagation in scattering media.^24^ These contributions highlight the importance of combining tailored detection geometries with physically grounded modeling and further motivate the development of approaches such as STOC that control coherence and scattering at the level of the measurement process.

### 1.3 Partial Spatial Coherence in *In Vivo* Imaging

Spatial incoherence is a well-characterized statistical property of light, and it is relevant in everyday visual perception under sunlight or incandescent or LED illumination. Remarkably, however, few studies have systematically examined how spatial partial coherence affects image reconstruction in strongly scattering media.^25^^–^^27^ This gap is especially evident in experimental setups that deliberately control spatial coherence and directly assess its influence on image formation in scattering objects.

To date, much of the literature has concentrated on exploiting spatial coherence as an additional gating mechanism, often referred to as spatial-coherence gating (sCohG).^4^ Indeed, widefield interferometric methods such as full-field optical coherence tomography (FF-OCT), holographic microscopy, and phase microscopy have demonstrated that illuminating a scattering sample with spatially incoherent light causes the interference contrast to diminish rapidly outside the focal region, thereby creating a “virtual gate” analogous to axial sectioning.^28^^,^^29^ This virtual spatial gate can deliver optical axial sectioning (OAS) with precision comparable to that achieved with tCohG, which is the standard in scanning systems such as OCT.^7^^,^^18^^,^^29^ Early FF-OCT configurations indeed exploited inexpensive, spatially incoherent light sources (e.g., LEDs or halogen lamps) to introduce tCohG at the micrometer scale; in these cases, sCohG emerged more as a passive by-product than a primary design goal. Pioneering work by Karamata et al. has deliberately leveraged spatial incoherence to suppress crosstalk and speckle in scattering media.^4^^,^^16^^,^^17^^,^^25^^,^^30^ Furthermore, a theoretical framework for FF-OCT imaging in turbid media has recently been developed to include the effects of partial temporal and spatial coherence, offering an explicit description of how these coherence properties shape the system’s optical transfer function and the depth-dependent signal decay.^27^

In practical implementations, researchers have adopted various strategies to modulate spatial coherence more actively. For example, using a long multimode optical fiber (MMF) to scramble spatial modes (TEMs) can reduce spatial coherence while preserving higher photon flux than thermal sources, thereby offering a compromise between brightness and coherence control. In such an approach, the intermodal delay between the supported TEMs exceeds the coherence length of the source, intermodal coupling is minimized, and partial spatial coherence is achieved.^31^ Experimental demonstrations illustrate the practical benefits of these techniques. Dhalla et al. described an improvement in the modulation transfer function (MTF) in FF-OCT when imaging through scattering media using a multimode fiber to reduce spatial coherence.^32^ They also investigated how reducing the spatial coherence areaby employing fibers with different core diameters and lengths (to values below the system’s lateral resolution)affects the rejection of multiply scattered photons through the formation of a spatial coherence filter in the FF-OCT system.^32^ In 2006, the effectiveness of partially coherent spatial illumination in a Fourier-swept OCT system was demonstrated, using a titaniumsapphire laser with an acoustic-optical filter and a short multimode optical fiber with active mode coupling attenuation *via* microbends at acoustic frequencies.^33^ Alternative approaches include rotating diffusers or pseudo-thermal light sources delivering diffuse quasi-monochromatic illumination. These methods have proven effective in enhancing image quality in scattering environments.^34^^–^^36^

Nonetheless, an inherent trade-off remains. Once spatial-coherence gating (sCohG) becomes comparable to or dominates the temporal-coherence gating (tCohG), the achievable imaging depth is often curtailed. For example, in full-field OCT systems, the entire input aperture is uniformly illuminated to maximize power from a halogen lamp or LED, resulting in complete spatial incoherence. As a result, many high-resolution reconstructions are limited to superficial tissue layers. In practice, overcoming this limitation tends to require sequential refocusing or dynamic focusing to maintain image quality at greater depths. This constraint compromises practicality, especially for *in vivo* imaging, and remains a fundamental bottleneck for coherence-based imaging methods such as FF-OCT in strongly scattering media.

### 1.4 Idea of STOC Imaging

The introduction of spatio-temporal optical coherence (STOC) imaging is based on the premise that high-order image distortions induced by light propagation through biological tissue can be suppressed by dynamically modulating the spatial phase of the illumination and integrating this approach with interferometric detection. Building on this concept, a method was formulated that achieves control of spatial and temporal coherence, designated STOC Imaging or STOC phase manipulation. When applied to volumetric datasets, this approach becomes STOC-tomography (STOC-T). Through averaging of interferograms recorded under distinct spatial-phase modulation masks, STOC preferentially preserves ballistic (or minimally distorted) photons while reducing the contribution of scattered photons. Multiply scattered photons (or, equivalently, optical waves whose wavefronts have been heavily perturbed) produce spatially randomized phase patterns; thus, under a variety of phase masks, their contributions average out, yielding negligible fringe visibility. By contrast, the undistorted wavefronts corresponding to mirror images interfere constructively across the phase-mask ensemble, enhancing fringe contrast. By further subtracting the DC component of the interference signal, image reconstruction relies exclusively on the variable (interferometric) part of the signal, thereby isolating contributions from the coherent light path. To further accentuate decoherence of distorted paths, the illumination is swept across multiple wavelengths in continuous mode while interferograms are recorded synchronously. This spectral-sweeping strategy not only amplifies the suppression of multiply scattered contributions but also enables full three-dimensional reconstruction *via* a temporal-coherence gating (tCohG) mechanism, analogous to that employed in Fourier-domain OCT.^9^

Earlier studies addressing speckle reduction and crosstalk suppression in OCT systems primarily relied on reducing the spatial coherence of the illumination. For example, Kim et al. demonstrated that the use of a partially spatially coherent source reduces speckle through statistical averaging of multiple independent realizations of the optical field. A similar concept was applied by Dhalla et al. in a parallel FF-OCT configuration, where the parameters of a multimode fiber were selected such that the radius of the spatial coherence area of the illumination at the sample was comparable or smaller than the system’s lateral resolution. Under these conditions, the illumination can be treated as locally spatially incoherent, effectively creating a spatial coherence filter that limits interference between signals originating from different points in the sample and thereby suppresses crosstalk associated with multiple scattering.

The concept of STOC and its extension STOC-T is based on a slightly different physical principle. Rather than driving the illumination toward full spatial incoherence, this approach relies on controlled temporal modulation of the spatial phase of the optical field while preserving interferometric detection. Crosstalk reduction is achieved by acquiring multiple interferometric measurements with different spatial phase realizations of the illumination. During volumetric reconstruction or acquisition, these measurements are averaged in such a way that ballistic contributions remain correlated across realizations, while signals originating from multiply scattered photons vary randomly and therefore cancel statistically.

Consequently, approaches based on reduced spatial coherence (Kim et al., Dhalla et al.) operate by locally suppressing interference, whereas STOC/STOC-T employs dynamic modulation of the spatial phase distribution combined with averaging, enabling high-quality volumetric imaging at significant depths in strongly scattering media. In practice, this difference is reflected in the radius of the spatial coherence gate, which in the works of Kim, Dhalla, and Povazay was on the order of a few to tens of micrometers, whereas in STOC-T it extends to the millimeter scalegiving axial gating comparable to scanning confocal OCT systems.

In this article, I present the detailed theoretical foundations underlying STOC and STOC-T, describe experimental realizations to date, and outline promising directions for future development and application.

## 2 Basics of STOC

According to the definition of the inverse problem in optics, the internal structure of tissue is reconstructed from measurements of scattered or reflected light, where the spatial distribution of the refractive index n is described by the function $F(r,k_{l}^{\left(i\right)})=-k_{l}^{\left(i\right)}(n^{2}(r)-1)$.^1^

Assuming monochromatic wave illumination and elastic scattering, the total field $E(r,k_{l}^{\left(i\right)})$ at a point in space designated by the position vector r is the sum of the incident and scattered electric fields, as follows:

$$E(r,k_{l})=E^{\left(i\right)}(r,k_{l}^{\left(i\right)})+E^{\left(s\right)}(r,k_{l}^{\left(s\right)}),$$ (1)

where the index l denotes the parametric dependence on wavelength. The solution to this problem takes the form of an integral equation that formally describes the sum of the elementary contributions of spherical waves generated by the incident wave $E^{\left(i\right)}(r,k_{l}^{\left(i\right)})$ at points determined by the position vector **r** of an object located at a distance $\left|r_{0}-r\right|$ from the detector [ Eq. (2)].^1^

$$E^{\left(s\right)}(r,k_{l}^{\left(i\right)})=\frac{1}{4\;\pi }{∭}_{-\infty }^{+\infty }F(r,k_{l}^{\left(i\right)})(E^{\left(i\right)}\left(r,k_{l}^{\left(i\right)}\right)-E^{\left(s\right)}\left(r,k_{l}^{\left(i\right)}\right))\cdot \frac{\exp\{-ik_{l}^{\left(s\right)}\cdot \left|r_{0}-r\right|\}}{\left|r_{0}-r\right|}d^{3}r.$$ (2)

Usually, optical imaging devices meet two additional conditions: far-field detection and backscattered-light registration, which allow Eq. (2) to be transformed to Eq. (3),

$$E^{\left(s\right)}(r,k_{l})=E^{\left(\mathrm{MI}\right)}(r,k_{l})+E^{\left(\mathrm{OC}\right)}(r,k_{l}).$$ (3)

Here, $E^{\left(\mathrm{MI}\right)}(r,k_{l})$ is an undisturbed field that contributes to the formation of a mirror image, as expressed by Eq. (4):

$$E^{\left(\mathrm{MI}\right)}(r,k_{l})=\frac{1}{4\;\pi r}{∭}_{-\infty }^{+\infty }F(r,k_{l})E^{\left(i\right)}(r,k_{l})\cdot \exp\{-i2k_{l}r\}d^{3}r.$$ (4)

In turn, $E^{\left(\mathrm{OC}\right)}(r,k_{l})$ is the optical crosstalk field; i.e., the field originating from wavefront disturbances associated with scattering in the sample [Eq. (5)]:

$$E^{\left(\mathrm{OC}\right)}(r,k_{l})=-\frac{1}{4\;\pi r}{∭}_{-\infty }^{+\infty }F(r,k_{l})E^{\left(s\right)}(r,k_{l})\cdot \exp\{-i2k_{l}r\}d^{3}r.$$ (5)

Equations (3) and (5) do not provide an analytic solution in the general case, because scattered-field components $E^{\left(S\right)}(r,k_{l})$ appear on both sides of the integral equation. This inherent nonlinearity underlies the interpretational difficulties of image signals acquired under optical-crosstalk (OC) conditions, which is a direct consequence of multiple scattering in the tissue. Fortunately, the OC field enters the recorded signal simply as an additive component to the total field. Under appropriate boundary conditions, this component can be neglected, thereby recovering the classical first Born approximation in which the incident field $E^{\left(i\right)}(r,k_{l}^{\left(i\right)})$ serves in place of the scattered field $E^{\left(s\right)}(r,k_{l}^{\left(i\right)})$ throughout the scattering volume.^1^ In practical terms, satisfying the Born approximation essentially requires that the scattered waves emerging from the sample preserve undistorted wavefronts and that the illumination can propagate without being affected by the object; such conditions rarely hold for complex, *in vivo* biological tissues. Indeed, these deviations strongly limit the class of specimens and imaging modalities amenable to quantitative 3D reconstruction *via* intensity-based imaging (e.g., classical cameras or microscopes).

**Fig. 1:**
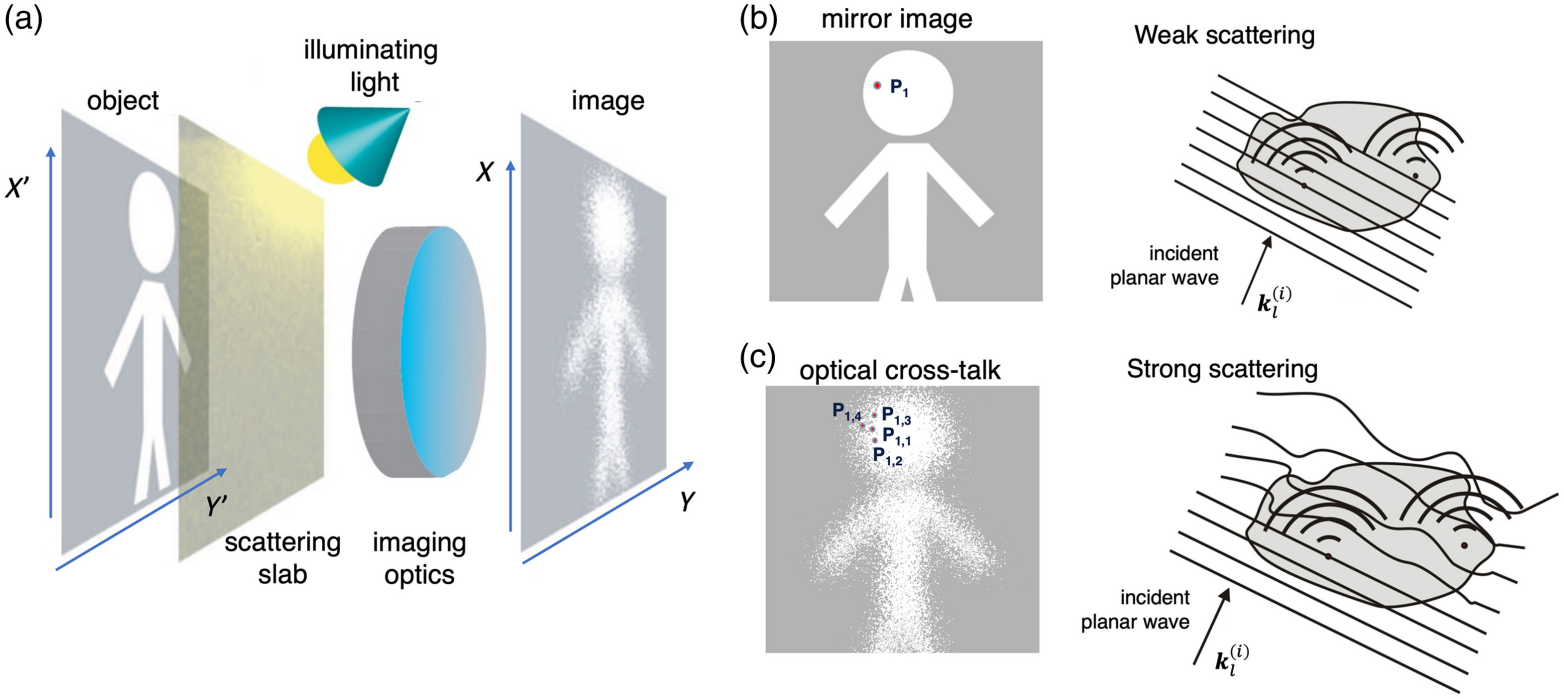
Schematic model of optical crosstalk (OC) formation. (a) Conceptual setup: a single reflecting plane is located beneath a diffusive slab. (b) Under ideal conditions (no scattering or only very weak scattering), light from point $P_{1}$ in the object plane maps directly onto the corresponding detector pixel, yielding a perfect mirror image, in which case the first-order (Born) approximation applies, and there is no crosstalk. (c) In a heterogeneous, scattering medium, this mapping breaks down; light scattered from a single object point contributes to signals recorded by many detector pixels, leading to optical crosstalk and loss of image fidelity.

Under typical *in vivo* conditions, only superficial layers (e.g., skin or other near-surface tissue) remain sufficiently undistorted to produce usable images. Moreover, inspection of Eq. (3) reveals that the only realistic way to suppress the disturbed (multiply scattered) contributions is to operate on the electromagnetic field itself, rather than on light intensity, because intensity measurements inherently mix mirror (ballistic) and scattered components. Consequently, any attempt to eliminate OC *via* purely numerical postprocessing, or even through deep learning applied to intensity images, is unlikely to succeed in general. By contrast, holographic or interferometric modalities, which retain information about the electromagnetic field (amplitude and phase), may offer a viable path toward effective filtering of scattering-induced distortions.

Intuitively, one associates optical crosstalk (OC) with the distortion of both the illumination and scattered optical waves; i.e., with the breakdown of the conditions underlying the Born approximation. Figure 1(a) illustrates this situation with a model experiment in which a perfectly reflective object lies hidden behind a scattering slab. When scattering is weak and the Born approximation holds, the system behaves like a perfect optical system; light re-emitted from a point $P_{1}$ on the object propagates undisturbed, and is detected at a unique corresponding point $P_{1}$ on the detector [Fig. 1(b)]. In contrast, under strong scattering, energy originating at $P_{1}$ is redistributed across many locations in the image plane, producing OC [Fig. 1(c)]. The reconstructed image becomes a superposition of the ideal mirror image plus contributions arising from waves whose wavefronts have been severely distorted. In such a configuration, the main goal for any depth is to recover the mirror-image component that represents the object’s structure. In principle, this recovery could be achieved by filtering out the scattered-field components responsible for OC, provided that a sufficient fraction of undistorted electromagnetic waves returns to the detector after attenuation. To formalize this notion, Eq. (3) can be generalized as the sum of two complex field contributions: the coherent (ballistic) component corresponding to the mirror image, and a scattered component representing the OC background, as shown by the following equation:

$$E^{\left(s\right)}(r,k_{l})=A^{\left(\mathrm{MI}\right)}(r,k_{l})\exp\{-i\Theta \}+\underset{n}{\sum }\underset{m}{\sum }[A_{n}^{\left(\mathrm{OC}\right)}(r,r_{m},k_{l})\exp\{-i(\Phi_{n}+\Phi_{m})\}].$$ (6)

We assume that the initially planar illumination wave may undergo n successive scattering events before arriving at the spatial point denoted by r, and simultaneously that m additional scattered waves from other locations within the object may also arrive at the detection point. As a result, the explicit amplitude $A_{n}^{\left(\mathrm{OC}\right)}(r,r_{m},k_{l})$ and phase $\Phi_{n}+\Phi_{m}$ of the total field become effectively nondeterministic [see Eq. (5)]. By contrast, the undisturbed (ballistic) field, arising from the initial planar illumination interacting with the object, produces a reconstructed image with a well-defined amplitude and phase distribution directly reflecting the object’s structure.

### 2.1 Spatial Phase Modulation as a Method of Introducing Partial Spatial Coherence

According to the coherence theory developed by Emil Wolf, a partially coherent light source can be formally represented as a superposition of mutually uncorrelated TEMs, each of which is fully spatially coherent in the spatial-frequency domain.^31^^,^^37^ In an experimental implementation, one can approximate generation of such uncorrelated TEMs by subjecting a coherent input beam to time-varying spatial-phase modulation *via* a spatial-phase modulator (SPM), as in Eq. (7):

$${\overset{˜}{E}}^{\left(s\right)}(r,k_{l},t)=E^{\left(s\right)}(r,k_{l},t)\;\exp\;[i\Delta \varphi (r,k_{l},t)],$$ (7)

where Δφ(r,t) denotes the phase shift introduced by the SPM. When successive phase masks are uncorrelated and the sample induces scattering, the component of the field arising from multiple scattering acquires random phase distortions for each mask $\mathrm{Rand}(\hat{\Phi }(r,k_{l},t))$ in the total scattered field, as expressed by Eq. (8):

E˜(s)(r,kl,t)=A(MI)(r,kl) exp {−iΘ+Δφ(r,kl,t)}+An(OC)(r,rm,kl) exp {−iRand(Φ^(r,kl,t))}. (8)

Suppression of these non-deterministic contributions is achieved by introducing a reference electromagnetic field that represents the original undistorted illumination wavefront (i.e., the “mirror-image” component). This reference is modulated by the same sequence of SPM masks [Eq. (9)]:

$${\overset{˜}{E}}^{\left(\mathrm{Ref}\right)}(r,k_{l},t)=E^{\left(i\right)}(r,k_{l},t)\;\exp\;[i\Delta \varphi (r,k_{l},t)].$$ (9)

By recording the interference between the scattered field and the reference field, the relationship shown by Eq. (10) is obtained.

$$I=\left\langle ({\overset{˜}{E}}^{\left(s\right)}\left(r,k_{l},t\right)+{\overset{˜}{E}}^{\left(\mathrm{Ref}\right)}\left(r,k_{l},t\right)){\left({\overset{˜}{E}}^{\left(s\right)}\left(r,k_{l},t\right)+{\overset{˜}{E}}^{\left(\mathrm{Ref}\right)}\left(r,k_{l},t\right)\right)}^{*}\right\rangle .$$ (10)

Mixed terms containing scattered waves, which carry random mask-dependent phases, average to zero over the ensemble of masks, effectively eliminating optical crosstalk (OC), as shown in the expressions of Eq. (11):

$$\langle {\overset{˜}{E}}^{\left(\mathrm{MI}\right)}(r,k_{l},t){\overset{˜}{E}}^{*(\mathrm{OC})}(r,k_{l},t)+{\overset{˜}{E}}^{\left(\mathrm{OC}\right)}(r,k_{l},t){\overset{˜}{E}}^{*(\mathrm{MI})}(r,k_{l},t)\rangle =0;\;\left\langle {\overset{˜}{E}}^{\left(\mathrm{Ref}\right)}(r,k_{l},t){\overset{˜}{E}}^{*\left(\mathrm{OC}\right)}(r,k_{l},t)+{\overset{˜}{E}}^{\left(\mathrm{OC}\right)}(r,k_{l},t){\overset{˜}{E}}^{*\left(\mathrm{Ref}\right)}(r,k_{l},t)\right\rangle =0.$$ (11)

By contrast, the coherent (ballistic) component carrying true object information remains consistent across the masks and therefore persists after averaging [Eq. (12)]:

$$\left\langle {\overset{˜}{E}}^{\left(\mathrm{MI}\right)}(r,k_{l},t){\overset{˜}{E}}^{*\left(\mathrm{Ref}\right)}(r,k_{l},t)+{\overset{˜}{E}}^{\left(\mathrm{Ref}\right)}(r,k_{l},t){\overset{˜}{E}}^{*\left(\mathrm{MI}\right)}(r,k_{l},t)\right\rangle =2A^{\left(\mathrm{MI}\right)}(r,k_{l})A^{\left(\mathrm{Ref}\right)}(r,k_{l})\cos\{-i\Theta )\}.$$ (12)

Although the phase modulation introduced by the SPM also perturbs the coherent image component [per Eq. (4)], these perturbations are systematic across all masks. Thus, after averaging over many uncorrelated phase masks, the artificially induced phase shifts cancel out, yielding a reconstruction that reflects only the undisturbed object wavefront and excluding contributions from scattering-induced distortions. This methodology, therefore, implements a filter that discriminates against scattering-induced OC by operating on the complex electromagnetic field rather than on intensity alone.

### 2.2 Construction of the G Coherence Matrix

**Fig. 2:**
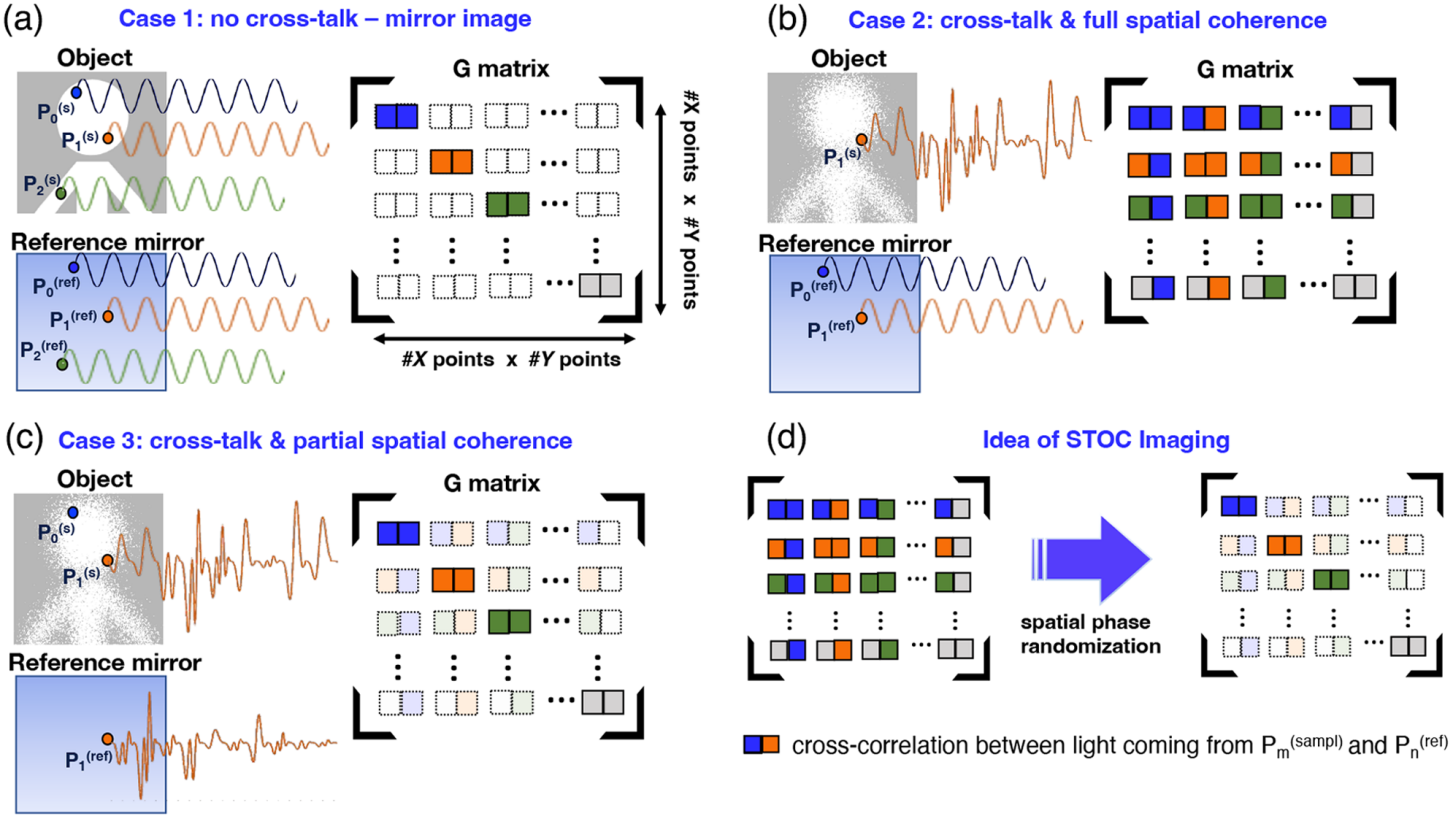
Schematic representation of the impact of scattering on the coherency matrix G. (a) Under ideal conditions (no scattering and coherent illumination), light from corresponding points on the object and the reference mirror maps directly onto matching detector pixels, producing a perfect mirror image without optical crosstalk. (b) When scattering occurs, parts of the wavefront deviate, causing optical crosstalk; i.e., signals collected by one detector pixel correlate strongly with those from other pixels, visible as nonzero off-diagonal elements in G. (c) If illumination is spatially decorrelated, waveforms recorded across detector pixels also become decorrelated; only pixels receiving identical temporal phase waveforms (i.e., from the reference mirror or mirror-component of object scattering) remain correlated. (d) By applying spatial phase modulation, spatial coherence is suppressed, so G becomes essentially diagonal and the image reconstruction remains undistorted. In the diagrams, sinusoidal waveforms illustrate coherent light with fixed phase relationships; irregular waveforms represent the temporal phase instability typical of scattered light. Axes X and Y correspond to the horizontal and vertical dimensions of the imaged object at a single imaging depth.

Based on this model, one can derive an algebraic formulation of the optical crosstalk (OC) effect that accurately reflects realistic experimental conditions. As discussed above, the main objective is to distinguish the “mirror-image” (ballistic) component of the field from the OC (scattered) component. To this end, a reference electromagnetic field representing the original, undisturbed wavefront illuminating the object is introduced. A point-by-point correlation is then performed between the temporal waveforms of the reference field and those of the disturbed field recorded from all object points. Because the reference field preserves the original phase and amplitude, this correlation enables the extraction of the mirror-image contribution, which is the only component that interferes with the reference. By contrast, scattered, OC contributions exhibit random phases and lack correlation with the reference and therefore do not contribute significantly to the interference signal.

This analysis further assumes that the fields are temporally stationary and ergodic. For simplicity of notation and without loss of generality, consideration is restricted to a single detection plane (e.g., the camera sensor plane) described in a Cartesian coordinate system $\left(x_{n},y_{m}\right)$. The two-dimensional detector array is further mapped onto a one-dimensional index through sequential enumeration of individual pixels [Eq. (13)]:

$$(x_{n},y_{m})\to \chi_{j=n+m*N},$$ (13)

where $x_{n}\in (0..N)y_{n}\in (0..M)$. With this model, it is most convenient to use the function of mutual coherence of time courses [Eq. (14)],

$$\Gamma (\chi_{j},\chi_{k},\tau )=\left\langle E^{*\left(s\right)}(\chi_{j},t)E^{\left(s\right)}(\chi_{k},t+\tau )\right\rangle .$$ (14)

The normalized correlation is represented by a complex degree of coherence, with magnitude ranges from 0 (no coherence) to 1 (perfect coherence), according to Eq. (15):

$$\gamma (\chi_{j},\chi_{k},\tau )=\frac{\Gamma (\chi_{j},\chi_{k},\tau )}{\sqrt{\Gamma \left(\chi_{j},\chi_{j},0\right)\Gamma \left(\chi_{k},\chi_{k},0\right)}},$$ (15)

where $\Gamma (\chi_{j},\chi_{j},0)$ is the autocorrelation function. The mutual correlations between each point in the reference field and every point in the object field can be expressed in algorithmic form as a matrix of complex coherence coefficients, hereafter referred to as the scattering-coherence matrix G [Eq. (16)].^38^^,^^39^

$$G=[\begin{array}{cccc} \gamma_{11} & \gamma_{12} & \cdots & \gamma_{1K}\\ \gamma_{21} & \gamma_{22} & \cdots & \gamma_{2K}\\ \vdots & \vdots & \ddots & \vdots \\ \gamma_{K1} & \gamma_{K2} & \cdots & \gamma_{KK} \end{array}].$$ (16)

In the matrix G, each element $\gamma_{kj}\equiv \gamma (\chi_{j},\chi_{k},\tau )$ quantifies the correlation between the fields detected at different pixels in a single detector plane. In addition, the matrix G is parametrically dependent on the light wavelength, indicated by the index l. By construction, G is symmetric (or Hermitian in the complex-valued case), and its dimensionality corresponds to the number of detector pixels, K=N·MxN·M. Figure 2 presents a schematic model of the matrix G, contrasting the case of optical recording without crosstalk to the case with crosstalk. In the ideal (no-OC) scenario depicted in Fig. 2(a), the reference field is provided by light reflected from a reference mirror.

When the undisturbed mirror image of the object and the reference image are projected onto the camera, only the mirror-image light can correlate with its counterpart from the reference field because pixels are mapped perfectly. Consequently, only the diagonal elements of G are nonzero, while all off-diagonal elements are absent. In the presence of OC and under spatially coherent illumination of a highly scattering sample [Fig. 2(b)], light scattered from a single object point is redistributed across multiple detector pixels [Fig. 1(c)]. These scattering yields significant correlations between the electromagnetic fields originating at different object points and the reference mirror, which are manifested as nonzero off-diagonal elements in G [Fig. 2(b)]. In this representation, off-diagonal (mixed) elements correspond to signals delocalized by scattering, whereas the diagonal elements represent undisturbed image points, i.e., ballistic photons returning with preserved wavefronts.

Finally, Fig. 2(c) illustrates how spatially uncorrelated illumination alters the structure of G. When each point across the beam cross-section carries a distinct, uncorrelated temporal amplitude and phase profile (a unique phase “fingerprint”), interference with the reference field only occurs for matching temporal profiles. Interference originating from light scattered at other object points induces random phase variations; as a result, after averaging, the amplitude of the interferometric signal due to disturbed (multiply scattered) field components vanishes. The coherence (or correlation) coefficient remains high only for undisturbed waveforms, namely, those corresponding to the same object point whose backscattered wave is reflected from the reference mirror. These undisturbed waveforms are the diagonal entries of the coherence matrix. As a result, off-diagonal elements are strongly suppressed, reflecting reduced coherence between scattered field components and the reference, which suppresses OC and preserves the fidelity of the reconstructed mirror image. In this way, the true “mirror image” of the object is recovered.

**Fig. 3:**
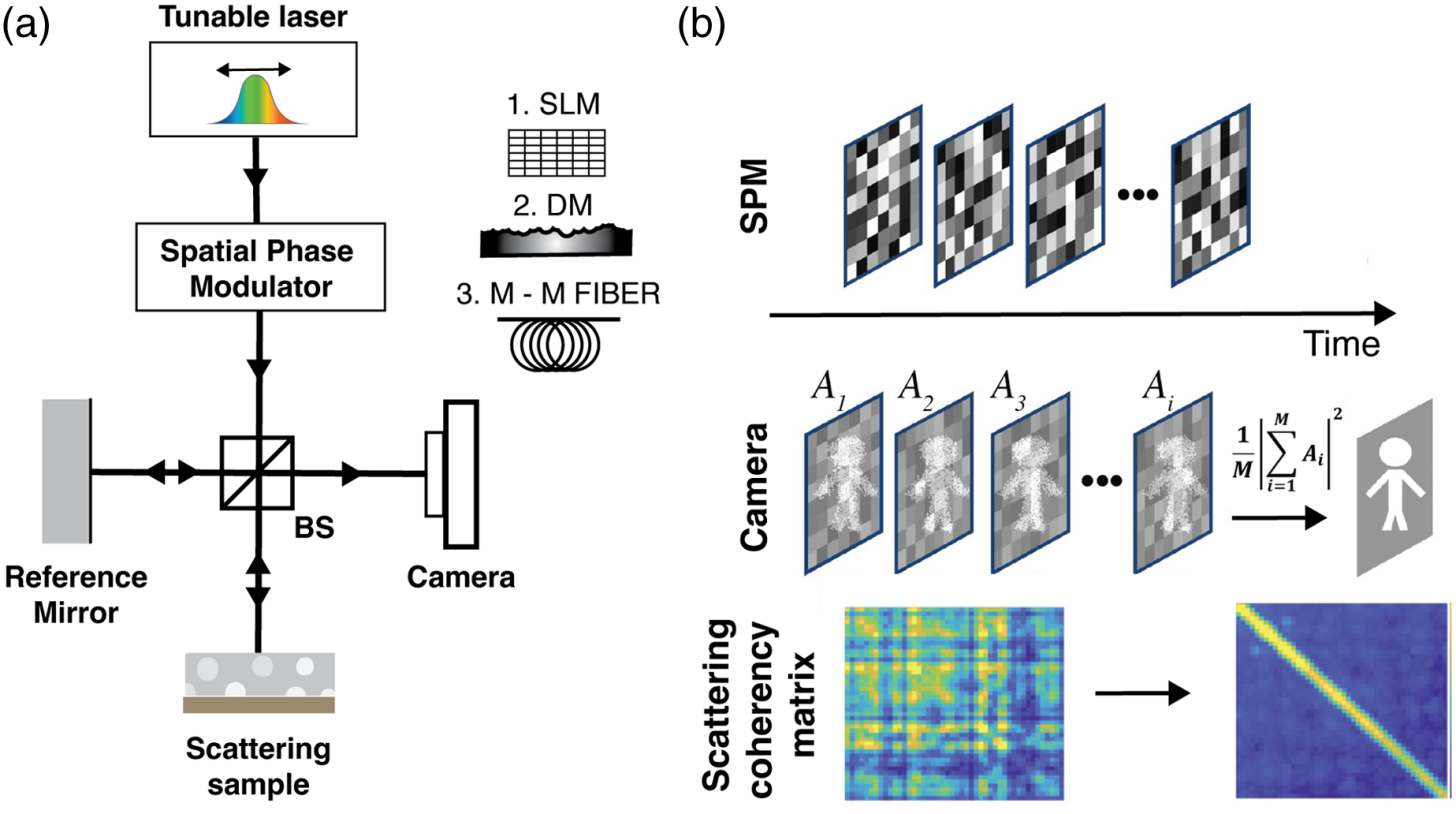
Visualization of spatial coherence *via* the scattering coherency matrix G: (a) Schematic of the basic STOC-T measurement setup. Three distinct approaches to spatial phase modulation were implemented and compared: via a spatial light modulator (SLM), via a deformable membrane (DM), and via a long multimode optical fibre; (b) Without application of STOC, spatial coherence remains high, as shown by prominent off-diagonal (nonzero) elements in scattering coherency matrix G. Note that low values fields correspond to image regions containing zero-value pixels and reflect data masking rather than genuine loss of coherence; when STOC manipulation is applied, spatial coherence is effectively suppressed, yielding a near-diagonal G matrix, consistent with strong spatial incoherence. SPM: spatial-phase modulator.

Practically, recovery of the mirror image from a scattering-distorted field reduces to averaging a set of interferograms acquired under many different, uncorrelated spatial-phase masks introduced by the SPM placed in the front of interferometer [Fig. 3(a)]. This averaging may be performed computationally after acquisition, or implemented in hardware by integrating multiple interferograms over a single camera exposure [Fig. 3(b)]. Crucially, success depends on the careful choice of spatial phase modulation across the beam cross-section. Only by selecting mask patterns (phase-mask shape, modulation amplitude, modulation rate relative to camera frame rate, etc.) that sufficiently “diagonalize” the coherence matrix can one guarantee robust suppression of OC and faithful reconstruction of the mirror image component [Fig. 3(b)]. In particular, the diagonalization of the coherence matrix in STOC is governed by parameters that reduce spatial correlations between detection channels, including the number and diversity of phase realizations, the strength of spatial phase modulation, and the applied averaging strategy. Orthogonalizing phase masks and operating on complete sets of variables (such as the set of Hadamard masks or modes excited in multimode optical fiber) promotes efficient decorrelation of multiply scattered components, which helps reduce off-diagonal elements in the coherence matrix. As a result, the matrix takes on a diagonal form, which directly translates to reduced crosstalk and improved image quality.^38^

### 2.3 STOC-Tomography (STOC-T)3D Reconstruction by STOC

In the considerations above, the image reconstruction was demonstrated only for a single object plane. In principle, however, STOC-based phase manipulation can be integrated into any interferometric or holographic setup (e.g., Doppler-laser holography, quantitative phase imaging, holographic microscopy, or similar modalities), depending on the intended application. This capability can also be extended to 3D imaging by employing polychromatic interferometric techniques, such as OCT. As previously noted, the theoretical framework easily generalizes to sequential light acquisition at different wavelengths. By capturing interferograms across a range of wavelengths (or optical frequencies), one obtains a volumetric data set of spectral interferometric fringes, akin to the approach used in Fourier-domain OCT methods. The implementation of STOC-T requires a wavelength-tunable light source synchronized with the camera’s acquisition. This approach mirrors the operational principle of full-field optical coherence tomography (FF-OCT), an interferometric technique that acquires *en-face* images with a camera rather than point-by-point scanning, delivering spatial resolution comparable to microscopy.^40^^,^^41^ Traditional FF-OCT operates in the time domain and typically uses spatially and temporally incoherent sources (e.g., arc lamps or LEDs),^42^ which helps reduce speckle noise and crosstalk, but the low power density of such sources, together with historically limited camera frame rates, has constrained *in vivo* application of FF-OCT to single axial depths.^43^ By contrast, Fourier-domain acquisition (as in FD-OCT) is inherently faster than time-domain scanning, which makes it an attractive match for the STOC concept, with the potential to reach imaging speeds suitable for *in vivo* volumetric imaging.^9^ In FD-FF-OCT implementations, the same advantages that make FD-OCT faster (i.e., Fourier-domain processing of spectral interferograms) can be combined with the wide-field camera-based *en face* acquisition of FF-OCT. ^44^^–^^47^ However, FD-FF-OCT inherently uses spatially coherent light sources, and because it lacks a confocal gating mechanism, it is prone to high levels of optical crosstalk.^4^^,^^45^ Therefore, introducing the STOC method into this imaging platform offers an attractive route to improve the quality of 3D reconstructions. Given the mechanism by which undisturbed image information is recovered under STOC, this approach is designated as STOC-T.^38^^,^^39^^,^^48^^–^^50^

### 2.4 Measurement and Analysis of the G-Matrix for Actual Scattering Objects

As described in preceding subsections, the reconstruction procedure required to achieve an undistorted image is fundamentally linear and entails generating a large ensemble of phase masks followed by averaging of the recorded interferograms. This averaging can be implemented in one of two principal ways:

1. by placing the spatial phase modulator (SPM) in the object arm of the interferometer, sequentially displaying a series of phase masks, simultaneously acquiring the corresponding interferograms, and subsequently averaging these recordings in postprocessing;
2. by positioning the SPM in front of the interferometer and rapidly cycling through phase masks such that the interferograms are averaged on the camera chip *via* temporal integration over the exposure time of the camera [Fig. 3(a)].

**Fig. 4:**
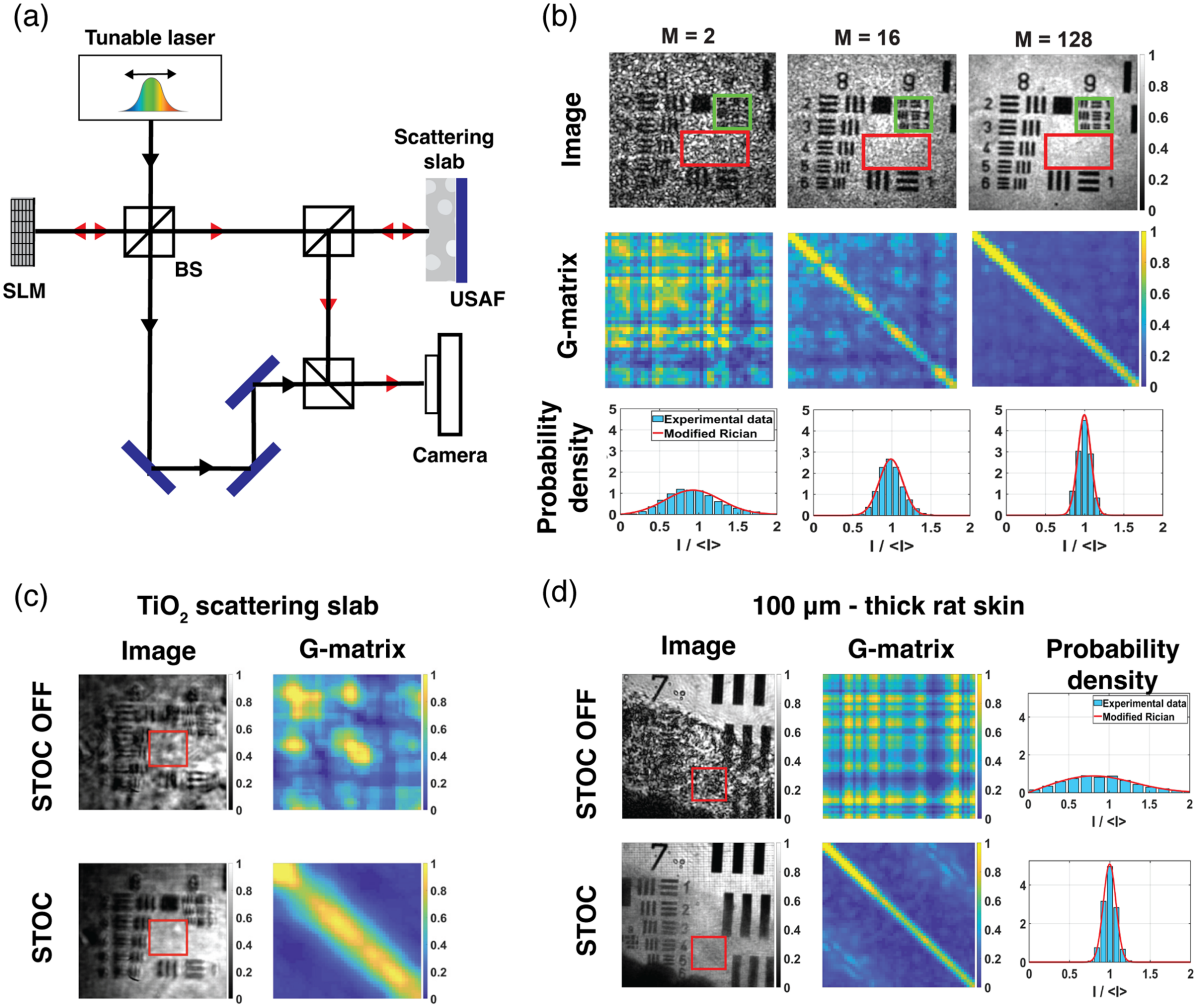
Characterization of spatial-coherence attenuation *via* STOC: (a) Schematic diagram of the optical setup employed for G-matrix measurements. (b) Control of spatial coherence through STOC is achieved by varying the number of applied phase masks, as evidenced by the enhanced contrast along the G-matrix diagonals and the corresponding changes in phase statistics; green rectangles in the intensity maps indicate regions used for G-matrix computation, whereas red rectangles denote areas used for phase statistical analysis. (c) Spatial-coherence modulation significantly enhances image quality in the presence of a ${\mathrm{TiO}}_{2}$ scattering layer positioned in front of a USAF-resolution test target. (d) A 100-μm-thick rat skin tissue layer introduces pronounced distortion in the reflected signal from the USAF target (left), whereas implementation of STOC reveals the underlying USAF structure; the resulting G-matrix exhibits clear diagonalization, confirming spatial-signal decoherence. Adapted from Ref. 38 with permission from Optica.

The reconstruction of the G-matrix necessitates the first strategy, because access to the temporal evolution of amplitude and phase for each pixel is required to compute inter-pixel correlations. The second strategy, while unsuitable for direct G-matrix reconstruction, may nevertheless facilitate practical implementation of the method for imaging applications. Figure 4(a) provides a schematic diagram of the measurement system used in this study, in which the SPM (in this case, a spatial light modulator, SLM) was placed exclusively in the object arm of a MachZehnder interferometer, following the configuration described in Borycki et al.^38^

In their study, they demonstrated the introduction of partial spatial coherence by imaging a USAF-resolution test target both without and with STOC modulation. Measurements were acquired using the optical system pictured in Fig. 4(a) and detailed in Borycki et al*.*;^38^ when STOC modulation was disabled, the resulting images exhibited characteristic artifacts associated with spatially coherent illumination, including pronounced speckle patterns around bars and numerals [Fig. 4(b)]. Enabling STOC manipulation suppressed these artifacts, attributable to the effective reduction in spatial coherence, a phenomenon corroborated by the diagonalization of the reconstructed G matrix. Moreover, as the number of averaged interferograms, and correspondingly the number of illuminating phase masks, increased, the contrast between the G-matrix diagonal and its background became increasingly pronounced.

Noise arising from crosstalk effects was quantified through statistical analysis, demonstrating that the STOC-T intensity probability density in the presence of crosstalk-induced noise conforms to a modified Rician distribution.^51^^–^^54^ This model describes the intensity distribution of the scattered field as the coherent superposition of a deterministic signal-phase vector and a random-phase vector arising from crosstalk-induced noise.^38^ In Fig. 4(b), the intensity images include a red rectangle delineating the region of interest (ROI) used for statistical evaluation. Within this ROI, the intensity of each pixel was normalized by the mean intensity ⟨I⟩, and the resulting distribution was fitted with a Rician probability density function [Fig. 4(b)]. With increasing numbers of phase masks, the normalized intensity distribution becomes progressively narrower and increasingly concentrated around ⟨I⟩. Consequently, the standard deviation of the crosstalk-induced noise $\sigma_{n}^{2}$ decreases, while the contribution of the undistorted signal $I_{d}$ grows, indicating that the useful signal increasingly dominates and the sample features become clearly resolved. Furthermore, STOC not only suppresses noise but also enhances spatial resolution; consequently, the line pairs of the ninth group in the resolution test target become distinguishable under STOC modulation compared to the unmodulated (STOC OFF) condition shown in Fig. 4(c). This improvement arises because coherent imaging systems are linear in complex amplitude; in contrast, incoherent systems are linear in intensity. From this perspective, the spatial resolution achieved with STOC-T is enhanced by a square root of two relative to the unmodulated, spatially coherent case.^38^

These preliminary experiments were designed to demonstrate the relationship between uncorrelated phase masks generated by the SLM and the attenuation of spatial coherence, as evidenced by the diagonalization of the coherence matrix. In the same study, Borycki et al. also reported measurements of a reflective USAF-resolution test target viewed through a current diffuser layer composed of titanium dioxide (${\mathrm{TiO}}_{2}$) embedded in polydimethylsiloxane (PDMS).^38^ With STOC manipulation disabled, the sample images are severely distorted [left column, Fig. 4(c)], and the corresponding scattering coherence matrix exhibits significant off-diagonal structure due to high spatial coherence, leading to pronounced crosstalk noise manifested by the presence of non-diagonal signals [right column, Fig. 4(c)]. Enabling STOC manipulation suppresses this noise, yielding images in which the test-target bars and numerals are resolved, and the scattering coherence matrix becomes predominantly diagonal, indicating effective reduction of spatial coherence [lower panels, Fig. 4(c)]. A qualitatively similar behavior is observed when a natural scatterer, in the form of a 100-μm-thick rat-skin layer, is placed atop the USAF target. Without STOC modulation, features beneath the scattering layer are obscured [right column, Fig. 4(d)], and the corresponding coherence matrices remain non-diagonal, with wide low-intensity distributions [middle and left columns, Fig. 4(d)]. When STOC is applied, the sample features are clearly recovered, spatial coherence is suppressed as indicated by the diagonalized coherence matrix, and the intensity distributions become narrow and high, confirming that the useful signal now predominates in the recorded measurement [Fig. 4(d)].

## 3 Methods

### 3.1 Basic Components of the STOC Optical Systems

As illustrated in Fig. 3(a), the STOC-T optical architecture comprises a tunable-laser source, a spatial-phase modulator, an interferometric arrangement, and a high-speed camera, supplemented by appropriate lenses and objectives, selected according to the imaging target (not depicted). All STOC implementations reported to date have employed a broadband swept-source laser (Broadsweeper BS-840-2-HP, Superlum) with an average output power of 25 mW and an instantaneous linewidth of ∼0.1  nm (coherence length <10  ps). The source enables continuous tuning over the 800 to 870-nm range with maximum sweep speeds approaching $1\times 10^{5}\;\mathrm{nm}/s$. For the experiments, the interferometric subsystem employed Linnik, Michelson, and Mach–Zehnder configurations. Interferometric recordings were acquired using a high-speed CMOS camera (Fastcam SA-Z, Photron, Tokyo, Japan) at 12-bit 512×512  pixel resolution, and up to a 60-kHz frame rate. Collectively, this instrumentation facilitated rapid multispectral interferometric image acquisition, a capability that was critical for volumetric data collection free of motion-induced artefacts.

**Fig. 5:**
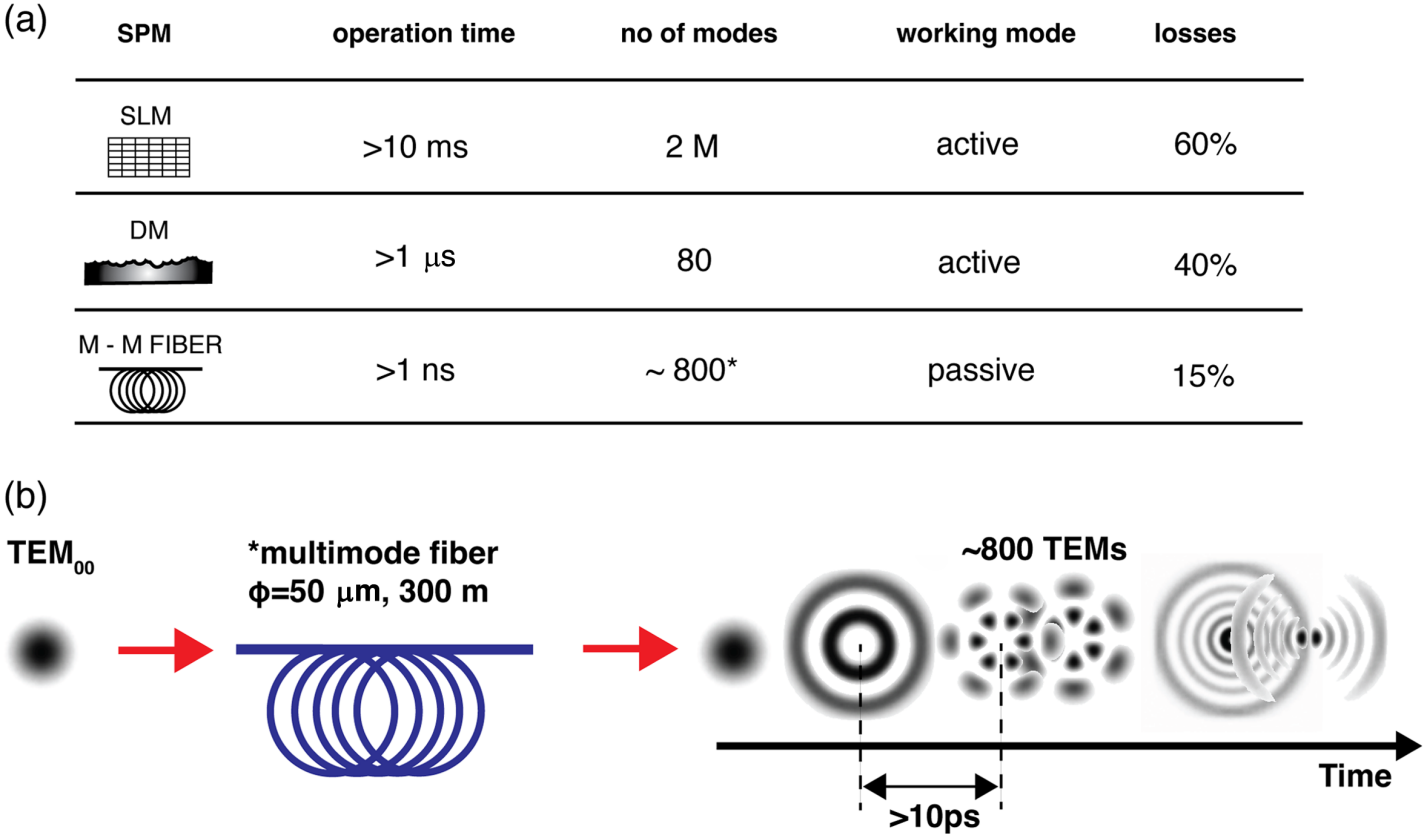
Summary of spatial-phase-modulation (SPM) operating parameters. (a) Table comparing three methods of introducing spatial phase modulation in STOC systems and their key parameters. (b) Schematic diagram illustrating phase decorrelation *via* generation of multiple modes in a long multimode optical fiber. The asterisk in panel (b) indicates that the number of modes listed refers to the step index multimode fiber configuration selected, with a core diameter of 50  μm and a length of 300 m.

A defining characteristic of all STOC implementations is the deliberate modulation of the spatial phase across the illumination beam to diminish spatial coherence. In practice, this modulation is effected by one of three mechanisms: (i) a liquid-crystal spatial light modulator (SLM, Holoeye PLUTO NIR2, 1920×1080  pixels); (ii) a deformable membrane (DM) with multiple piezo-actuators capable of up to $5\times 10^{5}$ chaotic oscillations per second (Dyoptica); or (iii) a multimode optical fiber, which decomposes a spatially coherent laser field into hundreds of propagation modes, thereby inducing partial spatial incoherence. Figure 5 summarizes the spatial phase modulators (SPMs) and key parameters relevant to the degree of induced partial spatial coherence. Among these devices, the SLM offers the greatest flexibility, providing full programmability of phase masks and, in the ideal case, on the order of 2-million degrees of freedom, enabling systematic exploration of the entire coherence space. However, the relatively low refresh rate of the SLM necessitates the sequential generation of hundreds of phase masks, resulting in volumetric data-acquisition times on the order of seconds. These temporal limitations, compounded by polarization control requirements and diffraction-related losses, render SLM-based modulation impractical for *in vivo* imaging applications. Nonetheless, the SLM remains an invaluable platform for validating the theoretical framework underlying STOC and for benchmarking key assumptions of the partial coherence hypothesis.^38^

An alternative approach to phase modulation employs a rapidly oscillating chaotic membrane actuator (Dyoptica) to impart uncorrelated phase patterns across the illumination beam at a rate that significantly exceeds the camera’s exposure time.^48^^,^^49^^,^^55^ In this configuration, the membrane’s high-frequency motion ensures that successive transverse-electromagnetic-mode (TEM) distributions are decorrelated over intervals longer than the coherence time of the swept source (i.e., >10  ps), thereby generating effectively uncorrelated phase masks during the integration period of each camera frame.^49^ This temporal decorrelation is essential for averaging the instantaneous phase variations toward a quasi-uniform distribution within a single exposure, which in turn reduces coherent artefacts in the recorded interferograms. The principal advantage of the membrane modulator is its substantially higher operational speed relative to liquid-crystal SLMs, enabling rapid generation of diverse phase patterns. However, this benefit is tempered by a limited number of actively excited TEMs (on the order of ∼80), which may be insufficient to fully eliminate OC. In addition, as with SLM-based modulation, the requirement for a reflective optical layout, optical losses associated with the modulator, and synchronization of the membrane motion with the camera acquisition impose further complexity on system design and alignment.

The final phase-modulation strategy was introduced by using a long multimode optical fiber as a passive mechanism for reducing spatial-coherence. In this arrangement, transverse electromagnetic modes (TEMs) are excited intrinsically through the propagation of the swept source light along the fiber, without the need for active modulation elements. For a step-index multimode fiber extended to lengths on the order of hundreds of meters, this passive excitation yields approximately 800 supported spatial modes at ∼300  m of fiber, as presented in Fig. 5(a).^50^^,^^56^^,^^57^ These modes become effectively decorrelated due to modal dispersion, which introduces intermodal delays exceeding 10 ps [Fig. 5(b)].^50^^,^^56^^,^^57^ For a fiber with a core radius a=25  μm and a numerical aperture NA=0.22, the number of guided modes at a wavelength of λ=840  nm is theoretically estimated to be approximately 840, according to the expression shown by Eq. (17):^56^^,^^58^

$$N\approx \frac{2\pi^{2}a^{2}{\mathrm{NA}}^{2}}{\lambda^{2}}.$$ (17)

Because crosstalk attenuation scales with the square root of N,^59^ this mode count corresponds to an expected reduction in crosstalk noise by a factor of approximately 29.

As previously demonstrated,^31^^,^^32^ further attenuation could be achieved by increasing the number of supported modes. However, unlike earlier studies employing long multimode fibers in OCT,^31^^–^^33^ in STOC, the resulting improvement must be balanced against the axial depth of field (DOF), which follows the relationship expressed in Eq. (18):

$$\mathrm{DOF}∼\frac{\lambda }{{\mathrm{NA}}_{\mathrm{ill}}^{2}},$$ (18)

where ${\mathrm{NA}}_{\mathrm{ill}}$ denotes the numerical aperture of the illumination. As ${\mathrm{NA}}_{\mathrm{ill}}$ increases with fiber-core radius and thus with the number of modes, enhanced crosstalk suppression comes at the expense of reduced axial range. The fiber length was selected to ensure sufficient temporal separation between individual modes, thereby preventing their mutual interference and suppressing speckle formation at the fiber output. The minimum fiber length required to achieve modal separation can be estimated according to Eq. (19):^56^^,^^58^

$$L_{\min}=\frac{4l_{c}\pi^{2}a^{2}n_{1}}{\lambda^{2}}=100\;m.$$ (19)

In practice, a 300-m-long fiber was employed, as the analytical expression does not account for degenerate spatial modes, which possess nearly identical propagation constants and group delays. Effective decorrelation of these modes necessitates substantially longer propagation distances. Compared with other phase modulation approaches, the long multimode fiber offers several advantages: it is the fastest modality because it requires no active control or phase pattern generation; it incurs minimal optical loss; and it does not necessitate synchronization hardware between the spatial-phase modulator and the imaging camera. These features make it particularly attractive for high-speed volumetric imaging, where coherence suppression is needed without complex control systems.

### 3.2 STOC-T Optical Configurations

**Fig. 6:**
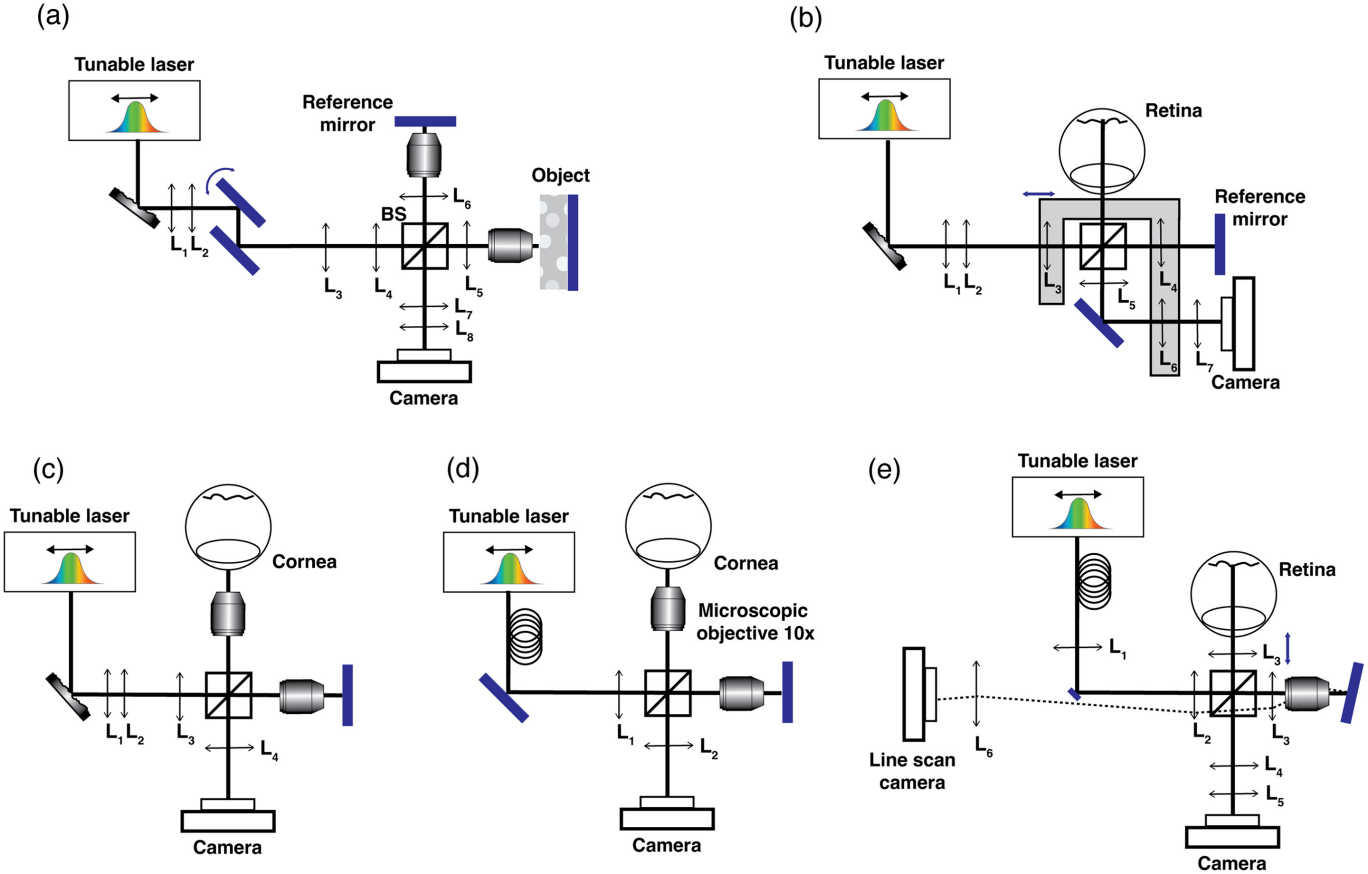
Schematic representations of optical configurations previously employed for STOC-T imaging. Systems incorporating a deformable membrane for *in vivo* imaging of (a) skin,^49^ (b) retina,^48^ and (c) cornea,^55^ and systems employing a 300-m multimode optical fiber for *in vivo* imaging of (d) the cornea^56^ and (e) the retina.^50^^,^^57^

Throughout the development of STOC and its volumetric extension STOC-T, a series of progressively refined measurement configurations was implemented to maximize performance across a range of experimental applications. Figure 6 depicts the schematic evolution of the optical system architectures employed in these implementations.

Figure 6(a) depicts the STOC-T system incorporating a deformable membrane (DM), which was applied for *in vivo* skin imaging and served as the first experimental platform for controlled spatial-coherence gating (SCohG).^49^ In this configuration, initial images of the DM surface are formed by a 4f relay comprising achromatic doublet lenses $L_{3}$ and $L_{4}$ (both with focal length f=75  mm). Subsequently, the reference mirror and sample were relayed by identical optical channels formed by achromatic doublet lenses $L_{5}$ or $L_{6}$ (f=50  mm) in conjunction with 10× microscope objectives (NA=0.25), arranged in 4f configurations. The sample and reference fields are recombined and projected onto the camera sensor through a final 4f relay consisting of achromatic doublets $L_{7}$ (f=50  mm) and $L_{8}$ (f=200  mm), yielding the desired system magnification. To maximize detection sensitivity, the optical magnification is selected such that the effective image scale on the sensor corresponds to 1.8×1.8  μm per pixel, implementing a double oversampling scheme that balanced optical and digital resolution. Angular compounding is introduced *via* a two-axis galvanometer scanner (Thorlabs GVS202, Newton, New Jersey, United States) to further suppress speckle contrast. In this module, the DM surface is imaged onto one of the galvanometer mirrors by a 4-f relay formed by achromatic doublets $L_{1}$ and $L_{2}$ (both f=50  mm), ensuring precise relaying of the deformable surface into the scanning assembly.

Figure 6(b) illustrates the first STOC-T system specifically engineered for retinal imaging,^48^ which likewise incorporates a deformable membrane (DM) for spatial-coherence control. In this configuration, long-focus relay lenses $L_{1}$ and $L_{2}$ again form a 4f relay that projects the DM onto a plane conjugate with the sample plane and subsequently onto the retina *via* the combined action of lens $L_{3}$ and the eye’s own optics. The reference arm comprises an achromatic doublet, $L_{4}$ (f=30  mm), serving as the objective, a reference mirror, and a neutral-density filter that attenuates the reference intensity to ∼6% in a dual-pass arrangement. Back-scattered light from the retina and the reference reflection are recombined and relayed to the detector through lenses $L_{5}$ and $L_{6}$, followed by tube lens $L_{7}$. To maintain sharp focus of the DM image on the retina over a range of refractive states, as well as on the reference mirror and camera plane, lenses $L_{3}$, $L_{4}$, and $L_{6}$ are co-mounted on a motorized translation stage for synchronous adjustment. During *in vivo* human eye imaging, the detection path magnification was approximately 10-fold, corresponding to an effective sampling interval of ∼2  μm on the retinal surface. Based on the system design and source characteristics, the axial resolution was estimated to be ∼4.5  μm.

An alternative STOC configuration incorporating a DM, specifically developed for *in vivo* imaging of the human cornea,^55^ is presented in Fig. 6(c). Owing to the relatively weak scattering properties of corneal tissue, system optimization in this case prioritized ocular safety rather than suppression of optical crosstalk. During imaging of the anterior segment, collimated illumination incident on the cornea is inherently focused by the eye onto the retina. The introduction of an actively modulated DM mitigates the risk of retinal photodamage by deliberately enlarging the retinal focal spot. In the system described in Auksorius et al.,^55^ the diffraction-limited spot diameter of ∼22  μm formes in the focal plane of lenses $L_{1}$ (f=50  mm) and $L_{2}$ (f=200  mm) is expanded by nearly a factor of 30. The enlarged focal plane is subsequently relayed and demagnified onto the pupil planes of identical objective lenses (UPlanFL N, Olympus; ×10, NA=0.3, FN=26.5) in both interferometer arms using relay lenses L2(f=200  mm) and L3(f=100  mm), producing collimated beams with a diameter of ∼1.2  mm that illuminate the cornea and the reference mirror.

Notably, the field of view remained unchanged following the reduction in spatial coherence, as the DM was optically conjugated with the sample plane. As a consequence of the coherence modulation, the effective retinal spot size increases approximately 30-fold (from 12  μm to ∼360  μm) ensuring safe retinal exposure for an incident corneal power of 5 mW. Back-scattered light is relayed onto a high-speed camera *via* a tube lens $L_{4}$ (f=300  mm), yielding an overall system magnification M=16.7.

A key advantage of architectures employing active spatial-phase modulation (SPM), such as those based on a DM, is the ability to switch seamlessly between operating modes. With the membrane inactive, the system operates under spatially coherent illumination, whereas activation of the membrane introduces partial spatial incoherence. This dual-mode capability enables direct, quantitative comparison of coherent and incoherent illumination regimes under identical experimental conditions.

The implementation of the spatial phase modulation (SPM) using long multimode fiber is illustrated in Figs. 6(d) and 6(e) for anterior segment (cornea/skin) and retinal imaging, respectively.^50^^,^^56^^,^^57^ Both configurations are markedly simpler to realize than earlier deformable membrane (DM) or spatial light-modulator (SLM)-based systems. In these implementations, a multimode fiber is coupled to a single-mode fiber at the output of a tunable laser source.^50^^,^^56^^,^^57^ The emerging laser emission is collimated and directed into a Michelson interferometer, where a nonpolarizing 50:50 beamsplitter divides the light between the sample and reference arms. In the corneal configuration, lens $L_{1}$ (f=100  mm), positioned upstream of the beamsplitter in conjunction with 10× objectives (UPlanFL N, Olympus; ×10, NA=0.3, FN=26.5), forms collimated illumination on both the sample and the reference mirror, yielding a symmetric Linnik interferometer. In the retinal configuration, the combination of lenses $L_{1}$ (f=50  mm) and $L_{2}$ (f=100  mm/75  mm for hi- resolution set-up) fulfil the beam-forming role, necessitated by the inclusion of a 45deg rod mirror for insertion of the viewing camera. In this geometry, lenses $L_{2}$ and $L_{3}$ (f=50  mm) image the distal tip of the single-mode fiber onto the pupil of the human eye and the reference microscope objective, respectively. Back-scattered light from the retina and the reference reflection (via 10x Olympus NA=0.25) are recombined and relayed to a high-speed two-dimensional camera *via* lenses $L_{4}$ (f=100  mm/75  mm) and $L_{5}$ (f=150  mm/300  mm). The resulting detection path magnification between the reference mirror and the 2D detector was configured to be×4 for the retinal system (10.5 in case of hi-resolution system)^60^ and ×16 for the corneal one via $L_{2}$ (f=300  mm). In the retinal setup, the portion of the interferometric signal not captured by the 2D camera, together with the reference beam, is directed to a line-scan camera (Alkeria Necta N4K-7-F) through lenses $L_{2}$ and $L_{6}$ (f=75  mm). The reference mirror is tilted by approximately 1deg to circumvent obstruction of the primary imaging path. This arrangement was adopted because the high-speed 2D camera could not sustain real-time transmission of B-scan images to the acquisition computer during patient alignment. By contrast, the line-scan detector, sampling one line at a time, enabled continuous real-time B-scan rendering for alignment and preview purposes.

### 3.3 Signal Acquisition and Processing

STOC-T acquisitions generate a sequence of 2D interferograms across a range of wavelengths (X, Y), typically capturing 512 frames per wavelength at an acquisition rate of 60 kHz. Each interferogram has spatial dimensions of 512×512  pixels. During acquisition, the laser wavelength is continuously tuned from 800 to 875 nm at a sweep rate of 8700 nm/s, yielding a single volume acquisition time of 8.6 ms. Between successive volume acquisitions, an idle period of ∼0.3  ms is required to reinitialize the laser tuning mechanism. Owing to limitations in dynamic range, 10 to 30 volumes per location were recorded, corresponding to a total recording duration of ∼90 to 265 ms. All data are subjected to post-processing. For each cross-section (X, Y), the DC component of the spectral fringe pattern is removed using a bandpass filter. In STOC-T, spatial filtering is applied to each X-Y plane to suppress low-frequency artefacts arising from fixed pattern noise. This procedure comprises a two-stage approach: estimation of the spatial filter followed by its application to the dataset, targeting specific spatial frequency bands. Filter estimation begins with a two-dimensional fast Fourier transform (FFT) of each plane, resulting in a 3D stack of complex values. Integration of signal amplitudes along the Z axis produces a 2D representation in which circular features corresponding to spurious signals are readily identifiable.^50^ The finalized spatial filter is then used to mask these frequencies selectively, and an inverse 2D FFT is performed at each Z depth to attenuate constant-pattern low-frequency noise. Subsequently, zero padding is applied to enhance axial sampling resolution digitally. A Fourier transform then yields volumetric complex representations (amplitude and phase) of the sample. Consistent with conventional Fourier-domain OCT, only one half of the transformed data is retained for further analysis due to the Hermitian symmetry of real signals in the Fourier domain.

To address chromatic dispersion mismatches between the interferometer arms and axial eye motion during laser scanning, an iterative correction algorithm adapted from prior work was employed.^61^ In STOC-T framework, the image quality metric is replaced with kurtosis, and the algorithm is extended to include five tunable parameters. Specifically, the composite signal is Fourier-transformed along the X-Z or Y-Z planes (B-scans) and multiplied by a parameterized phase corrector.^50^ Following the inverse Fourier transform, the sharpness of the resulting B-scans is quantified *via* the kurtosis metric. The phase coefficients are iteratively adjusted to maximize this sharpness measure, and the optimized phase factor is subsequently applied to all B-scans within the volumetric dataset.

To mitigate blurring due to defocus, a split-aperture correction technique as detailed by Giner et al.^62^ was employed. Starting with *en face* images, the data are transformed into the spatial-frequency domain using a two-dimensional fast Fourier transform (FFT). The resulting Fourier spectrum is partitioned into two equal sub-apertures, each of which is separately inverse-transformed. After estimating the relative displacement between the paired sub-aperture images, this procedure is repeated across multiple depths, and the resulting displacements are fit with a linear model. The fitted parameters are then used to derive a phase correction function expressed in Zernike polynomials. This correction is applied consistently across all volumetric datasets.

A key advantage of parallel interferogram acquisition is the inherent capacity to also correct higher-order aberrations.^63^^,^^64^ As it was demonstrated by Borycki et al., ^65^ this is achieved using an iterative optimization approach: the complex *en face* input image is first transformed via a two-dimensional Fourier transform, and the result is modulated by a variable phase mask composed of weighted Zernike polynomials. The modulated spectrum is then inverse Fourier-transformed to yield phase-corrected data. An image-sharpness metric based on entropy, computed from the intensity of the corrected image, is used to assess quality.^65^ This sequence of transformations and metric evaluation is repeated iteratively until the entropy measure is maximized.^50^^,^^65^

## 4 Results–Key Features of STOC-T Imaging

### 4.1 Optical Crosstalk Reduction

**Fig. 7:**
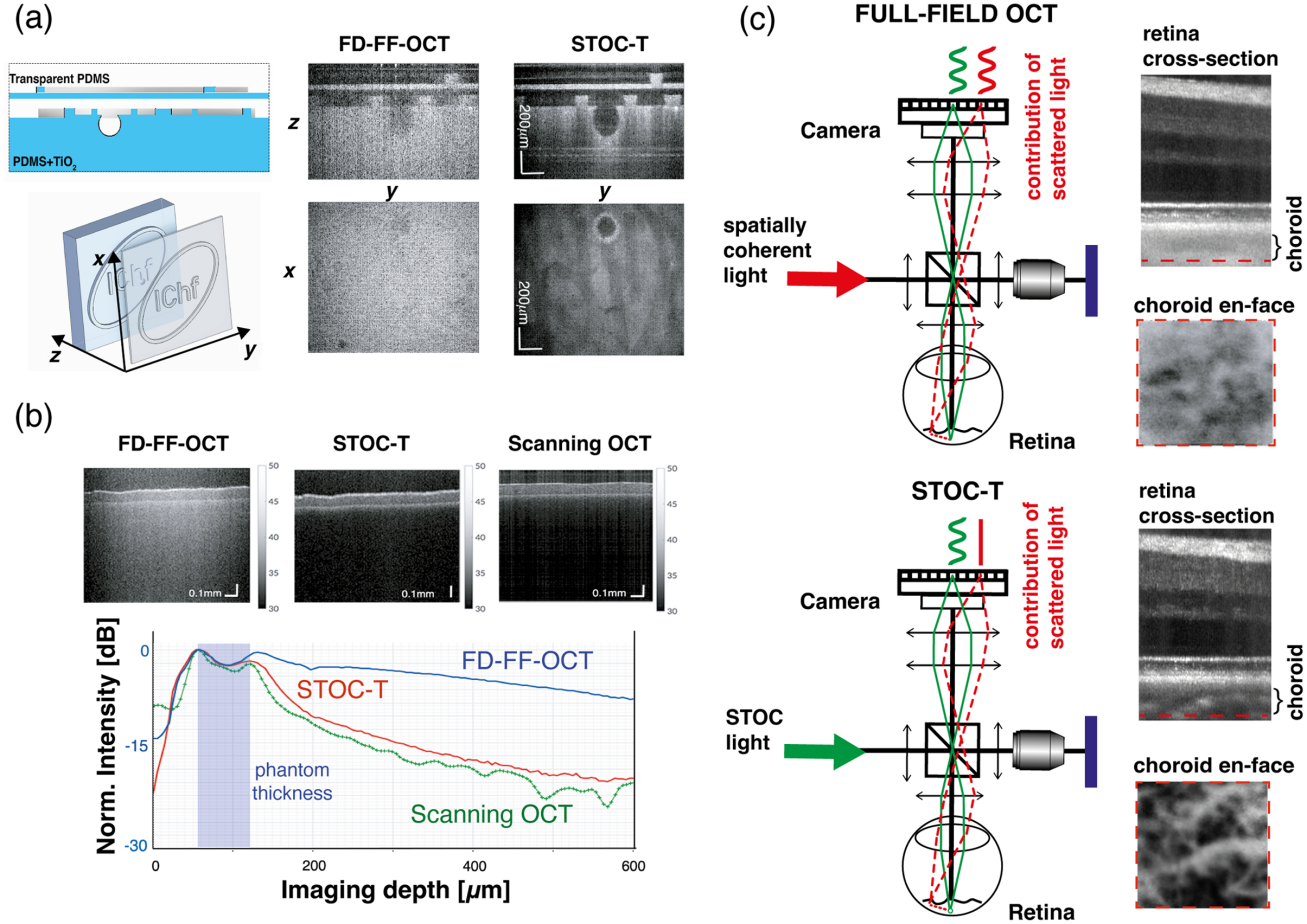
Optical crosstalk reduction with STOC-T imaging. (a) Measurement results for a thin latex phantom; (b) comparison of optical-crosstalk effects in STOC-T versus FD-FF-OCT images of a thick, scattering two-layer phantom; (c) Illustration of the impact of phase modulation, showing side-by-side fragments of *in vivo* retinal cross-sectional images acquired with and without spatial-phase modulation. Figure adapted from Refs. 49, 50, and 57 with permissions from Optica and CellPress licensed under CC-BY-4.0.

A principal advantage of the STOC-T architecture is its capacity to mitigate optical crosstalk arising from strong sample scattering through rapid modulation of the illumination’s spatial phase. In STOC-T, multiply scattered light that has lost its original spatial structure does not contribute to the interferometric signal or to the volumetric reconstruction. Studies cited here have demonstrated effective crosstalk suppression over an extended axial imaging range, using dynamic spatial-phase randomization implemented with SLM, rapidly deformable membranes, or long multimode fibers.^49^^,^^50^^,^^55^^–^^57^^,^^60^^,^^65^^,^^66^ The initial demonstration of OC minimization employed a two-layer $\mathrm{PDMS}/{\mathrm{TiO}}_{2}$ phantom, as described earlier [Fig. 7(a)].^49^ Reconstructions obtained with FD-FF-OCT exhibited an anomalously uniform signal throughout the phantom, inconsistent with Lambert–Beer attenuation, which predicts increased intensity loss for light traversing thicker scattering regions and should therefore yield contrast variation with depth. By contrast, STOC-T reconstructions revealed expected attenuation along longer photon paths, manifested as “shading” and discernible projections of the double-layer phantom structure. Moreover, a subtle spherical defect (∼180-μm diameter) within the phantom (barely detectable in FD-FF-OCT) was clearly resolved with high contrast using STOC-T, as quantified by Stremplewski et al.^49^

Further experimental validation of crosstalk suppression using long multimode fibers is presented in Fig. 7(b). A thin latex phantom (∼100-μm thick) was imaged with STOC-T, FD-FF-OCT, and a scanning OCT system; and depth-dependent signal-decay curves were derived by summing all A-scan lines. These curves demonstrate that STOC-T reduces crosstalk to levels comparable with scanning OCT systems employing a confocal aperture defined by a single-mode fiber. An example of optical crosstalk removal in *in vivo* imaging is shown in Fig. 7(c), which compares cross-sectional retinal images acquired with and without crosstalk suppression. Although structures anterior to the retinal pigment epithelium (RPE) are similarly resolved in both full-field OCT and STOC-T (with active deformable membrane), the photoreceptor layer and RPE exhibit markedly enhanced contrast after crosstalk removal. Furthermore, activation of the deformable membrane in STOC-T revealed choroidal morphology that remains obscured in conventional FD-FF-OCT images.

### 4.2 Enhanced Imaging Depth while Preserving High and Uniform Transverse and Axial Resolution.

**Fig. 8:**
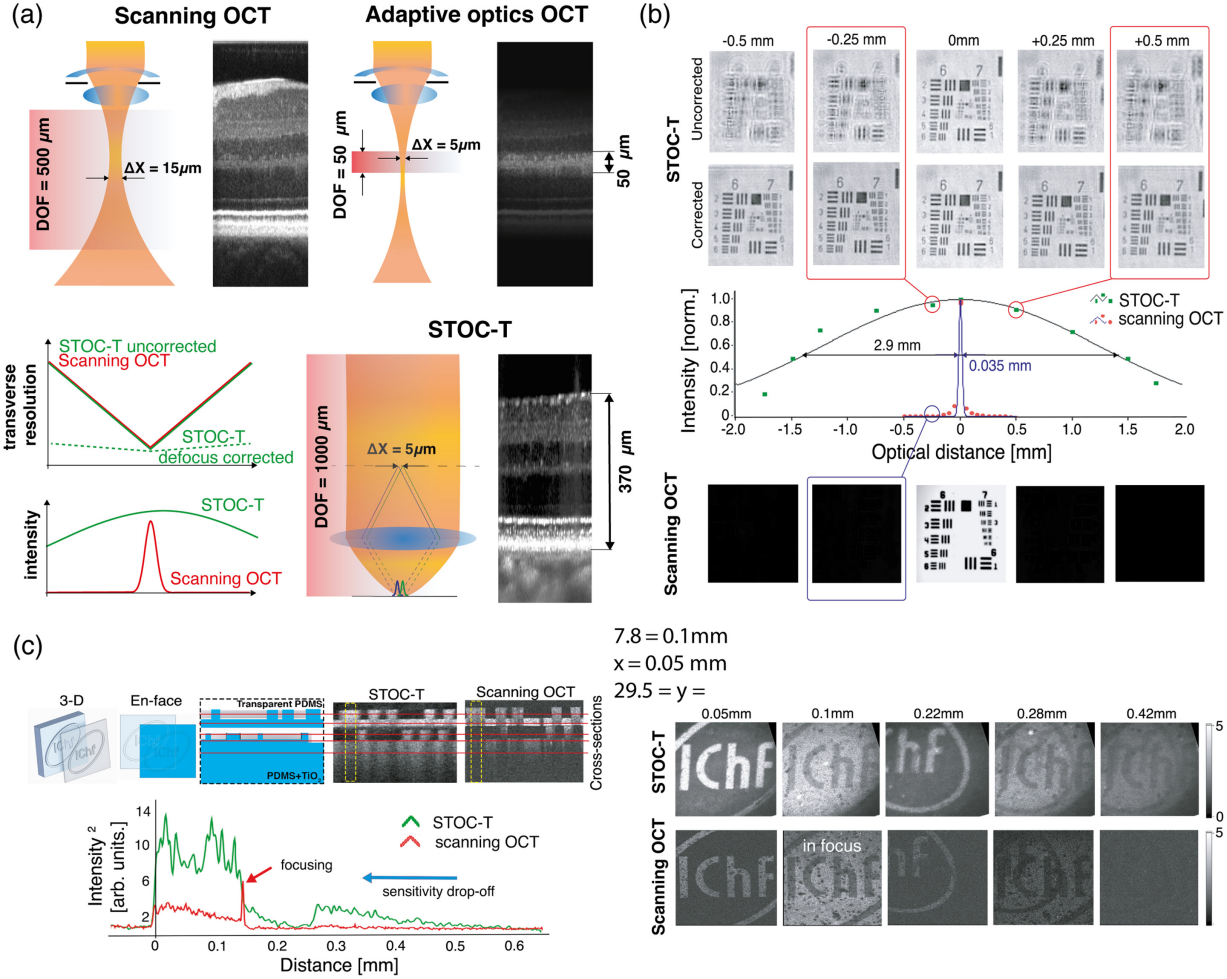
Comparison of depth-of-focus (DOF) performance in STOC-T and scanning OCT at matched lateral resolution. (a) Schematic diagram of illumination-beam formation together with cross-sectional images of a biological sample, illustrating differences in DOF between regular scanning OCT, AO-OCT, and STOC-T. (b) OCT imaging of a reflective USAF-resolution target acquired at increasing defocus, with additional numerical refocusing applied in the STOC-T reconstructions. (c) Depth-dependent signal attenuation measured in a volumetric-scattering phantom. Red lines in the cross-sectional images denote the axial locations of the corresponding *en-face* projections shown on the right, while the attenuation curves were extracted from the regions indicated by dashed yellow squares. Figure adapted from Ref. 50 licensed under CC-BY-4.0.

High-axial-resolution scanning imaging systems, such as OCT and confocal microscopy, inherently exhibit a limited confocal-imaging range, leading to an asymmetry between axial and lateral resolution.^9^ In scanning OCT, the axial-imaging range is constrained by the depth of focus (DOF), limited to the Rayleigh range of the focused beam, and both image sharpness and signal intensity fall off rapidly outside of this region as DOF decreases with improving lateral resolution [Fig. 8(a)].^50^ By contrast, STOC-T relies on full-field detection, which maintains signal intensity regardless of defocus. Through controlled spatial coherence, achieved by underfilling the illumination pupils in both interferometer arms, the technique enables imaging with an extended depth of field. Although defocus still induces image blur, this effect can be effectively compensated by numerical refocusing, allowing high lateral resolution to be sustained at increased imaging depths (see paragraph Sec. 3.3). Figure 8(a) presents a comparison of retinal images acquired with matched lateral resolution using STOC-T and scanning OCT. Although scanning OCT with adaptive optics (AO) shows localized signal enhancement constrained by its shallow DOF, STOC-T produces a uniform signal across the entire depth range for the same lateral resolution. This behavior is quantitatively evidenced in axial translations of a reflective USAF-resolution target [Fig. 8(b)], where STOC-T exhibits a DOF approximately two orders of magnitude greater than that of scanning OCT. In scanning OCT, defocusing the focal plane by 250 and 500  μm reduced the target signal to ∼1% and ∼0.5% of its peak (>−40  dB loss), respectively,; whereas in STOC-T, the same defocus diminished the signal only to ∼95% and ∼90% (<−1  dB), respectively, with residual blur largely correctable over ±1.5  mm *via* numerical refocusing.

In a controlled scattering experiment using a two-layer $\mathrm{PDMS}/{\mathrm{TiO}}_{2}$ phantom [Fig. 8(c)], the depth-resolved intensity profile of scanning OCT reveals a pronounced focal peak and rapid attenuation outside the focal plane.^49^^,^^50^ By contrast, STOC-T maintains object signal throughout the phantom thickness, with attenuation consistent with Beer–Lambert decay.

*En face* sections further demonstrate the superior contrast uniformity achieved with STOC-T relative to scanning OCT. To minimize sensitivity roll-off in the scanning system, measurements were configured with zero optical path difference located within the phantom. Both imaging modalities employed identical ×10 objectives and a nominal lateral resolution of 3.4  μm.

### 4.3 High Quality Volumetric Imaging with Isotropic Resolution

**Fig. 9:**
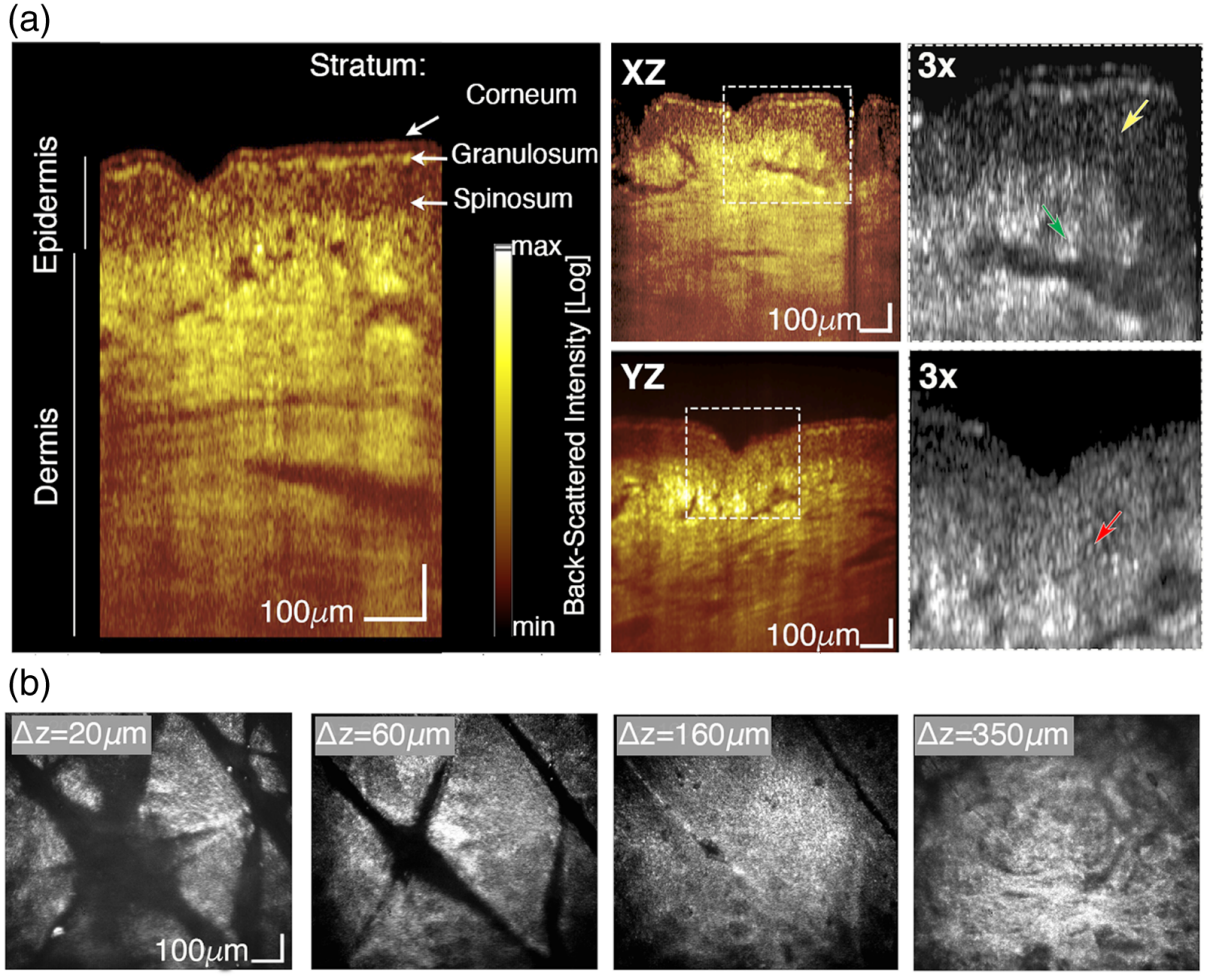
STOC-T imaging of *in vivo* human forearm skin. (a) Representative cross-sectional images. Yellow arrows mark granular cellular structures within the stratum spinosum, red arrows indicate putative individual melanocytes, green arrows denote sebaceous glands located in the dermis, adjacent to hair follicles. (b) *En face* projections of the epidermis and dermis extracted at selected axial depths, where Δz denotes the distance below the skin surface. The complete volumetric dataset (512×512×512 voxels) was acquired in 0.4 s (36 volumes). Figure adapted from Stremplewski et al.^48^ with permission from Optica.

A major strength of STOC-T volumetric imaging is its ability to deliver nearly isotropic micrometer-scale resolution within a 1×1×1  mm imaging volume—a capability that is currently unparalleled among *in vivo* tissue-imaging modalities. Such resolution enables the acquisition of high-fidelity cross-sectional images in all orthogonal directions. As illustrated in Fig. 9, cross-sections extracted from the same 3D dataset of *in vivo* forearm skin reveal structural features with ∼2.5-μm resolution, including distinct layers of the epidermis such as the stratum corneum, stratum granulosum, and stratum spinosum.^49^ The multilayered keratinocytes of the stratum spinosum are particularly well resolved. Hyper-reflective spots corresponding to individual melanocytes [red arrow, Fig. 9(a), inset] and sebaceous glands adjacent to hair follicles [green arrow, Fig. 9(a), inset] are also discernible. Beyond cross-sectional detail, STOC-T offers a clear advantage over conventional scanning OCT in the quality of frontal (*en-face*) reconstructions. This superiority is exemplified by the representative surface images of skin depicted in Fig. 9(b), which demonstrate enhanced contrast and structural clarity in the *en-face* plane.

**Fig. 10:**
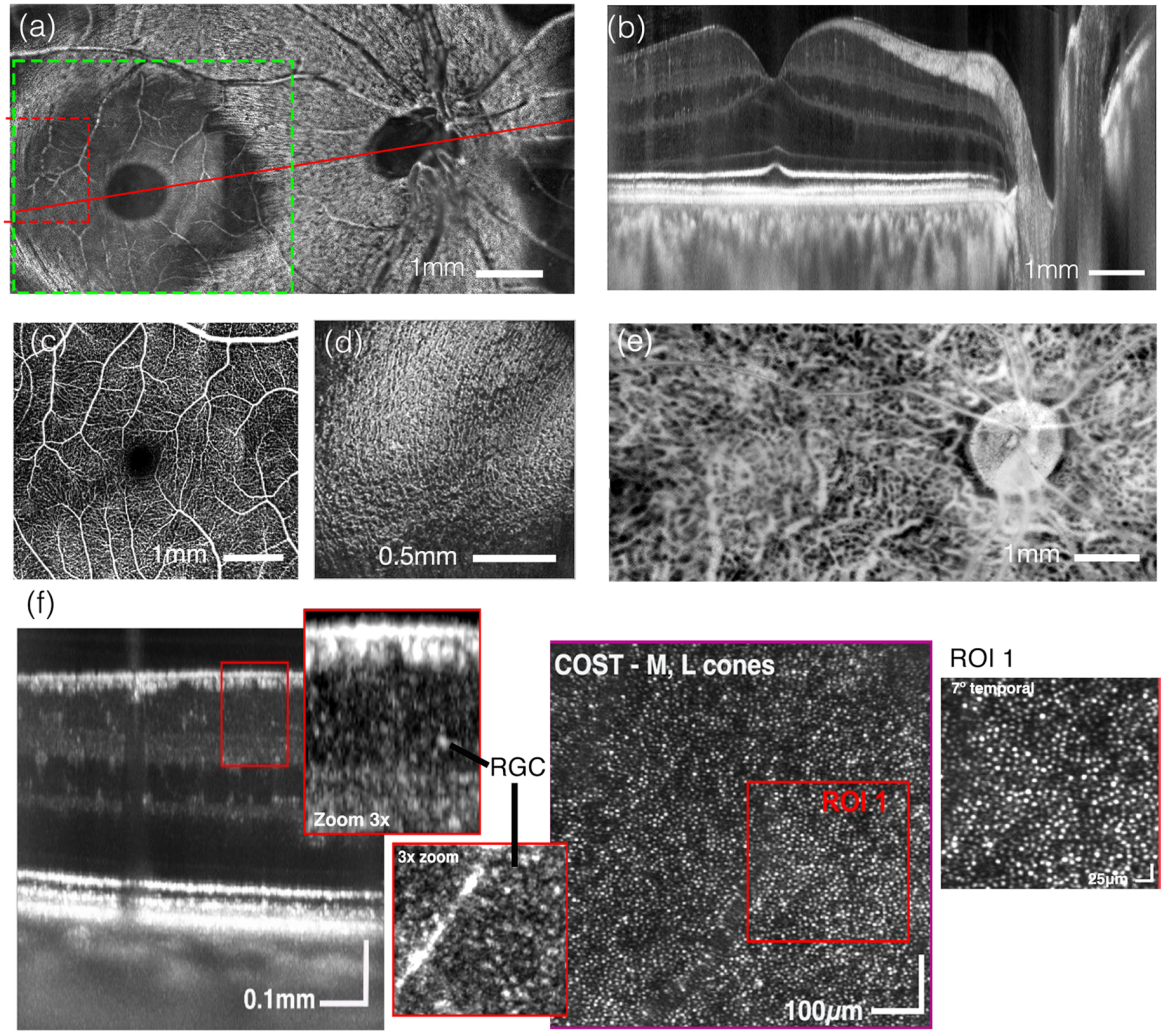
*In vivo* STOC-T imaging of the human retina-choroid complex. (a) *En-face* projection of the inner retinal layers at the level of the retinal nerve-fiber layer (NFL) and ganglion cell layer (GCL). (b) Axial cross-section of the human retina along the fovea–macula axis, as indicated by the red line in (a). (c) Angio STOC-T of retinal vasculaturethe region of interest marked with a green box in (a). (d) Detailed view of the hyloid membrane; the region corresponds to the red box, marked by the dashed line in (a). (e) Cross-sectional image of Sattler’s layer derived from a 20-μm-thick projection at a depth of 55  μm below Bruch’s membrane (contrast inverted). (f) High-resolution *in vivo* STOC-T imaging of retinal cells: cross-sectional (B-scan) view with a highlighted 3× zoom region showing retinal ganglion cells (RGCs) in both B-scan and en-face perspectives; plus an en-face projection of cone mosaics from the COST layer. Figures adapted from Auksorius et al.^50^ licensed under CC-BY-4.0 and our previous work^60^ with permision.

In ocular imaging, *in vivo* visualization of deeper structures remains a substantial challenge.^50^ As illustrated in Fig. 10, the limited imaging range of conventional confocal-scanning OCT precludes simultaneous high-resolution imaging of both the retina and the choroid. As discussed earlier in this chapter, STOC-T overcomes this constraint by employing full-field detection without optical crosstalk, combined with numerical correction of defocus and aberrations. This strategy enables high-quality volumetric reconstruction of retinal and choroidal structures across an extended depth range. Panels 10(a), 10(b), 10(d), and 10 (e), adapted from *iScience*,^50^ demonstrate the capability of STOC-T for simultaneous *in vivo* imaging of the human retina and choroid. *En face* [Fig. 10(a) and 10(e)] and axial [Fig. 10(b)] views were obtained by combining 32 three-dimensional STOC-T volumes into a composite field of view, measuring 9×4.6  mm. Notably, this dataset provides, for the first time, high-quality visualization of the hyloid membrane (HM), which delineates the nerve fiber layer (NFL) from the vitreous. Figure 10(d) displays resolved fine-structural details of the HM, which become less discernible in regions where vitreous contact and nerve fiber contributions dominate. The transition between the HM and the NFL is clearly visible throughout the montage.

The choroid exhibits pronounced structural complexity at multiple depths [Fig. 10(e)]. Owing to the relatively long exposure times associated with STOC-T volumetric acquisition, regions of elevated blood flow appear hyperreflective relative to the surrounding static choroidal tissue. To enhance visualization of the vascular architecture, the contrast of the *en face* projections was inverted, rendering low-reflectivity vessels highly reflective. The corresponding cross-sectional image [Fig. 10(i)] further reveals fine retinal features, including cone outer segments, which are only weakly visible in conventional scanning OCT images.

Figure 10(c) shows an example of angio-STOC-T reconstruction of retinal vasculature with visible both capillary plexuses and superficial retinal blood vessels.^67^ Furthermore, Borycki et al. ^68^ have shown that STOC-T datasets can be exploited for blood flow analysis by applying laser Doppler flowmetry principles to the Doppler broadening induced by moving erythrocytes. Unlike conventional laser holographic Doppler velocimetry, their approach employs multiple illumination wavelengths during acquisition, enabling the concurrent extraction of both three-dimensional structural information and blood flow contrast from a single STOC-T dataset. This dual capability has been formalized as multiwavelength laser Doppler holography (MLDH), which effectively integrates multiwavelength holographic detection with Doppler flow analysis to achieve high-speed, high-resolution *in vivo* visualization and potential quantification of microvascular flow across distinct retinal layers. The photoreceptor mosaic and retinal-ganglion-cell soma are further resolved in panels Fig. 10(f) through numerical-aberration correction when the highly resolution system with optimized SNR described by Zdankowska et al. is used.^60^

A key distinction from hardware-based adaptive optics is that the performance of computational approaches for correcting ocular defocus and aberrations is strongly contingent on the signal-to-noise ratio. When blurring reduces image contrast to the extent that structural information is buried in noise, accurate reconstruction becomes infeasible. This limitation motivates the use of hybrid strategies, such as precompensating refractive error with an adjustable lens placed in front of the eye to minimize defocus,^50^ or, as more recently demonstrated,^69^ integrating hardware adaptive optics with computational aberration correction.

In corneal imaging, the acquisition of high-quality microscopic *en face* sections is essential, as such views are of direct clinical relevance in corneal diagnostics. Traditionally, these images are obtained using contact confocal microscopy (e.g., Confoscan),^70^ which requires physical contact with the eye, local corneal anesthesia, and increased patient cooperation, thereby prolonging the examination. STOC-T, as a non-contact modality, provides an attractive alternative to these tactile techniques. As discussed earlier, full-field illumination with spatially coherent light poses a retinal safety risk because the collimated beam illuminating the cornea is focused onto the retina and may exceed permissible exposure limits.

**Fig. 11:**
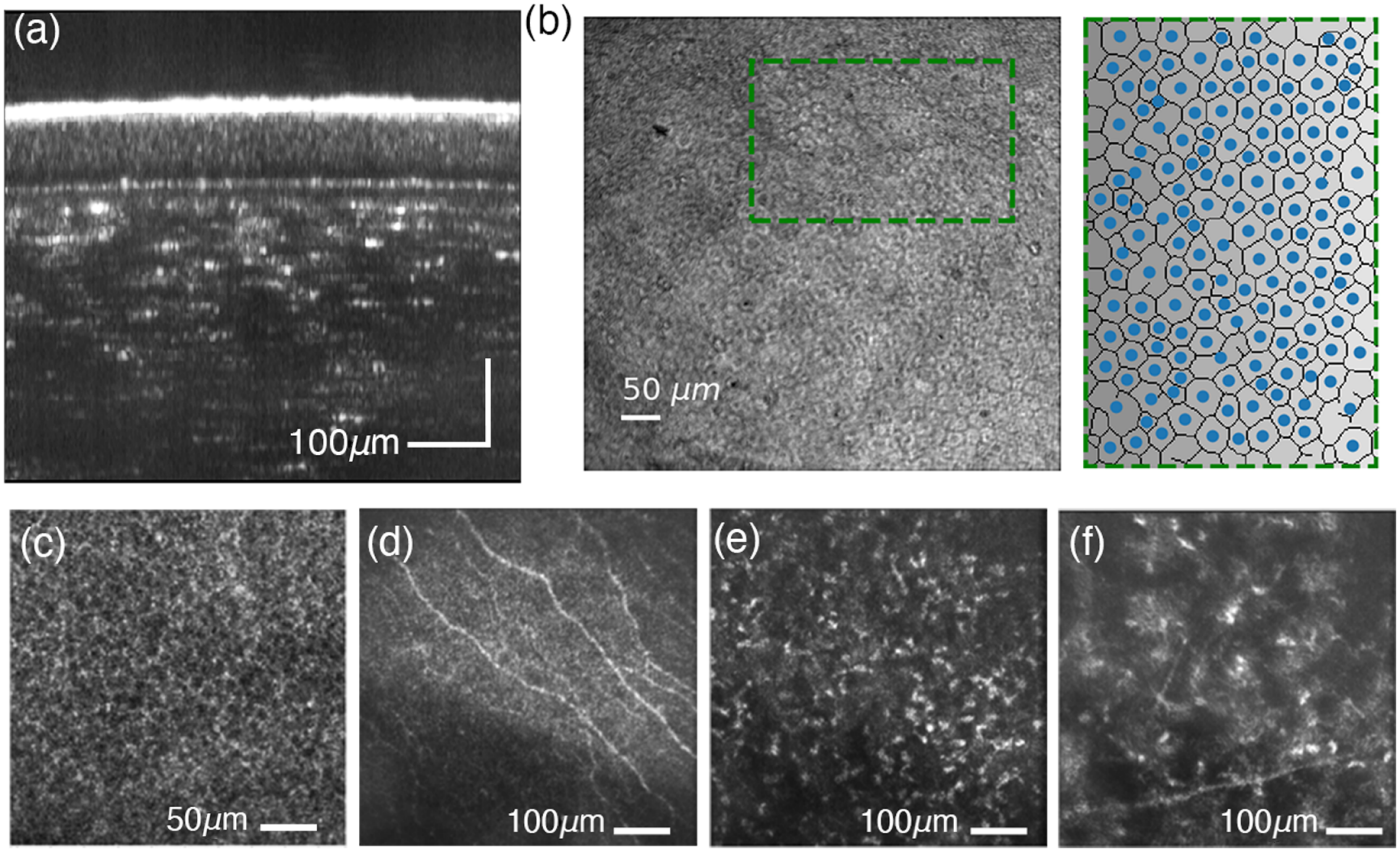
*In vivo* STOC-T imaging of the human cornea. (a) High-resolution cross-sectional view of the anterior cornea, resolving the epithelial layer and cellular morphology. (b) *En-face* image of the corneal endothelium with automated cell segmentation. (c) *En-face* image of the corneal epithelium acquired ∼40  μm below the surface. (d) *En-face* visualization of the sub-basal nerve plexus at a depth of ∼80  μm. (e) *En-face* image of the central stroma at ∼110  μm depth. (f) *En-face* image of the posterior stroma at ∼170  μm depth. Figure adapted from Auksorius et al. ^55^^,^^56^ with permission from Optica.

In STOC-T, this risk is mitigated by employing spatially incoherent illumination, which broadens the retinal focal spot and reduces local irradiance to safe levels. Using this approach, Auksorius et al. demonstrated that volumetric datasets acquired within fractions of a second enable high-fidelity reconstruction of a substantial portion of the corneal volume [Fig. 11(a)].^55^^,^^56^ The resulting images clearly resolve multiple corneal layers, including the endothelium [Fig. 11(b)] the epithelial cell layer [Fig. 11(c)], the sub-basal nerve plexus [Fig. 11(d)]. Following numerical correction of aberrations in the three-dimensional data, cellular-level corneal microstructure became clearly discernible. Epithelial cells were resolved at depths of 30 to 50  μm below the surface, subepithelial nerves at ∼80  μm, and stromal nerves at ∼170  μm. Keratocytes were observed in both the central [Fig. 11(e)] and posterior stroma [Fig. 11(f)]. In addition, STOC-T datasets were segmented for endothelial cell density and morphometric analysis, using a neural network trained on STOC-T and mirror microscopy images. The resulting morphometric parameters showed good agreement with those obtained using conventional specular microscopy [Fig. 11(b)].^70^

### 4.4 Functional Retinal Imaging Optoretinography (ORG)

**Fig. 12:**
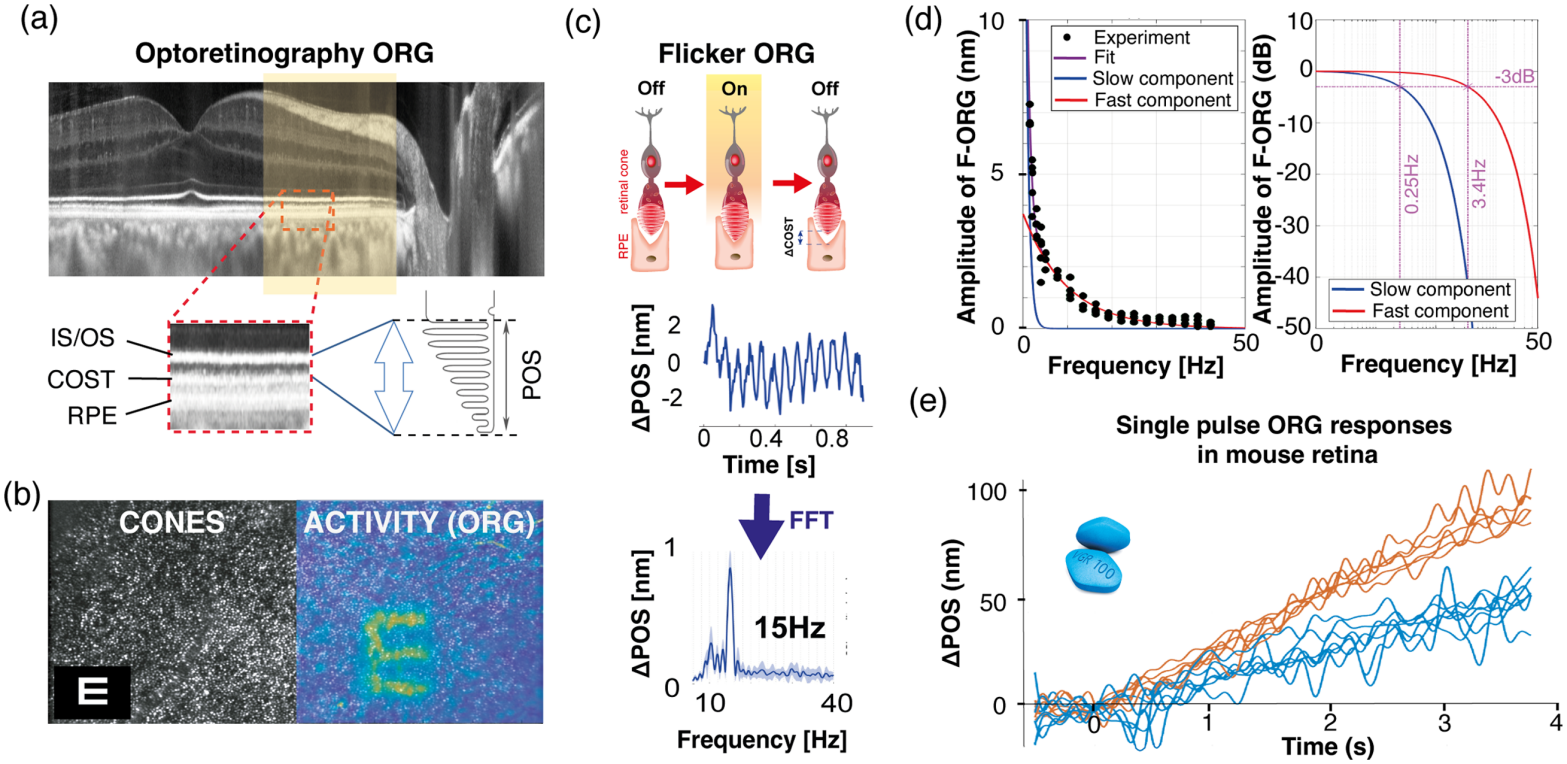
Optoretinography (ORG) measurements using STOC-T. (a) Schematic of ORG: light stimulation elicits nanometer-scale optical length changes in the photoreceptor outer segments (POS). (b) Presentation of an “E” optotype on the retina evokes responses in specific photoreceptors, which are localized by ORG and overlaid on STOC-T photoreceptor mosaics after computational aberration correction. (c) Flicker ORG (f-ORG) principle: the phase difference between bright IS/OS and COST layers in the STOC-T signal oscillates harmonically under light adaptation; subsequent Fourier transformation enhances signal-to-noise ratio. (d) Spectral profiles of cone responses to flicker stimulation from multiple STOC-T f-ORG recordings. (e) Single-pulse ORG traces under scotopic conditions in mouse retina before (orange) and after (blue) sildenafil treatment. Panel (c) data is reproduced from Tomczewski et al.^76^ with permission from Optica. Results presented in panels (d) and (e) are adapted from Tomczewski et al.^77^ licensed under CC-BY-4.0.

Optoretinography (ORG) refers to a number of techniques that measure light stimulus-evoked internal optical signals from groups of even single photoreceptor.^71^ Twenty years ago, it was shown that OCT could detect small changes in reflected light intensity occurring after retinal light stimulation *in vitro*^72^ and in animals *in vivo.*^73^ These findings laid the foundations for OCT-based ORG.^71^^,^^74^^,^^75^ A breakthrough in OCT-based ORG happened in 2016 when Hillmann et al. used an Fd-FF-OCT system to measure the photoreceptor response to a light stimulus in humans *in vivo.*^46^ Their results showed that the change in optical thickness of photoreceptor outer segments (ΔPOS)optical path length between photoreceptor inner/outer segment junction (IS/OS) and cone outer segments tips (COST) [Fig. 12(a)] in response to a stimulus consists of two phases: a rapid ΔPOS contraction (by single nanometers), followed later by a slower positive expansion of greater amplitude (hundreds of nanometers). Other important ORG results have been obtained using scanning OCT systems, often combined with adaptive optics. The slower positive expansion was explained in 2017, as resulting from diffusion of water into the outer segment of the retina to maintain osmotic balance during the phototransduction cascade.^78^

In 2019, it was shown that it is possible to use cellular-resolution ORG [Fig. 12(b)] to classify individual cones in humans *in vivo.*^79^ The following year, a high-speed adaptive optics line-scanning OCT system was used for cell-resolution ORG,^80^ and additional models were developed to explain the potential origins of both fast and slow responses.^81^^–^^83^ ORG has also been used to detect photoreceptor dysfunction *in vivo* due to retinal degeneration in mice^75^^,^^84^ and in humans.^85^ A publication by Pfaffle et al.^86^ demonstrated the ability to discriminate ORG signals for three different sublayers in the Inner Plexiform Layer of retina (IPL). Although the interpretation of the results obtained in this work is not clear-cut. It should be noted, however, that in all of the papers mentioned above, the observed effects were the result of experiments for scotopic vision (dark-adapted eyes). Moreover, most of them analyzed slow responses, which are more related to the adaptive function of the retina rather than to the visual transduction process itself. Consequently, these limitations in experimental design impose many interpretive and experimental constraints and open more questions than they clarify.

The introduction of the STOC-T system has enabled new capabilities for investigating the ORG phenomenon by combining high acquisition speed with enhanced contrast in the photoreceptor layer, yielding more robust and stable phase measurements of light-evoked responses.^66^^,^^76^^,^^77^ This improved performance facilitates the detection of rapid retinal dynamics that were previously inaccessible. To clarify the link between ORG signals and the underlying physiological response of the retina, flicker optoretinography (f-ORG) was developed as an approach that uses periodic light stimulation to capture rapid, light-evoked changes in photoreceptor function and dynamics under photopic (light-adapted) conditions [Fig. 12(c)].^66^ It has been shown that STOC-T is enabled to perform the reproducible and quantitative measure of rapid f-ORG in the human eye *in vivo* [Fig. 12(c)].^66^ To comprehensively characterize the ORG phenomenon, the frequency response of oscillatory photoreceptor signals was measured across the 1.5 to 45 Hz range using a chirped flicker stimulus and STOC-T imaging.^76^^,^^77^ The resulting frequency profiles exhibited double-exponential behavior, analogous to the dynamics observed for a single, dark-adapted ORG pulse,^87^ and are presented on linear and logarithmic scales in Fig. 12(d). Analysis of the −3  dB cut-off frequencies yielded low- and high-frequency components at ∼0.4 and 3.7 Hz, corresponding to time constants of ∼398  ms and ∼43  ms, respectively. Comparison with electrical recordings of photoreceptor voltage responses to sinusoidal stimulation obtained via patch-clamp in fresh primate retinas demonstrates close correspondence with L-M cone kinetics at light intensities below ∼5000  R*/s. This concordance supports the interpretation that STOC-T f-ORG signals directly reflect the local voltage response of cone photoreceptors.

To elucidate photoreceptor dynamics in response to transient light stimulation and to identify mechanisms underlying stimulus-evoked changes in photoreceptor length, controlled *in vivo* experiments were conducted using sildenafil, an FDA-approved inhibitor of phosphodiesterase 6 (PDE6),^88^^,^^89^ a key effector in phototransduction. Robust and sustained PDE6 inhibition required systemic doses of sildenafil exceeding established human safety thresholds and known to be photoreceptor-toxic, as reported by Yanoga et al.^90^ Albino mice (BALB/c) were chosen to facilitate high-quality optical imaging despite weaker visual responses relative to pigmented strains. Figure 12(e) shows a single-pulse scotopic ORG recordings from 12 representative datasets before and after sildenafil administration. Sildenafil treatment induced a marked attenuation of photoreceptor ORG signals. Quantitative analysis demonstrated a statistically significant reduction in scotopic ORG responses following PDE6 inhibition compared with baseline. These functional deficits were corroborated by concurrent assessments of visual pathway integrity using visual evoked potentials (VEP) and retinal imaging. These findings indicate that stimulus-induced elongation of photoreceptor outer segments, as measured by spatiotemporal optical coherence tomography (STOC-T), reflects conformational changes in PDE6 during phototransduction. The unique architecture of the photoreceptor outer segment amplifies these molecular-scale (ångström) conformational transitions into measurable cellular-scale (nanometer) structural changes that can be further measured by the STOC-T method.

Similar to photopic flicker electroretinography (ERG), photopic flicker optoretinography (f-ORG) using STOC-T holds substantial clinical translational potential, as the rapid characterization of photoreceptor frequency responses under light-adapted conditions within seconds may yield complementary biomarkers of retinal function and dysfunction. The practical advantages of the f-ORG paradigmincluding markedly shorter measurement durations and the elimination of prolonged dark adaptation and dark clinical environments - further favor its earlier clinical application compared with other ORG implementations.

## 5 Conclusion

In this contribution, I described spatio-temporal optical coherence imaging (STOC) and its three-dimensional extension, STOC-T *in vivo* imaging modality capable of high-resolution visualization within strongly scattering media without a priori knowledge of the medium’s optical heterogeneity. STOC achieves this through engineered phase manipulation across the beam cross-section combined with illumination by partially coherent light sources. Presented in literature studies demonstrate that sequential phase modulation, synchronized with data acquisition, effectively decorrelates coherent noise arising from spatial crosstalk. The crosstalk suppression is quantified via a scattering coherence matrix, and it has been shown that integrating either image intensity or complex amplitude further enhances image quality by reducing residual crosstalk artifacts. Compared with existing *in vivo* imaging technologies, STOC-T offers several compelling advantages: extended imaging depth with preserved lateral resolution, owing to a reduced reliance on confocal gating; improved lateral resolution enabled by effective utilization of larger pupil apertures; quantitative morphometry, facilitated by high-contrast volumetric reconstructions; enhanced phase stability, permitting sub-micron axial displacement measurements, including functional photoreceptor assessments in optical response-guided (ORG) mode and effective compensation of optical aberrations of objects or optical systems.

These advantages were validated through high-resolution, high-contrast *in vivo* imaging of retinal and choroidal microstructures, including choroidal capillaries that have historically been challenging to visualize due to their geometric thinness, low intrinsic contrast, and speckle noise. Relative to state-of-the-art optical coherence tomography (OCT), STOC-T decouples imaging depth from lateral resolution, achieving an extended axial range of ∼1  mm with an effective lateral resolution of ∼5  μm.

An intrinsic strength of the STOC paradigm is its hardware simplicity. In its simplest implementation, a multimode fiber acting as a phase modulator, only a basic interferometric setup and a high-speed CMOS camera are required. By emphasizing computational processing over complex hardware, STOC-T readily leverages advanced GPU computing and a rich suite of digital holography-derived algorithms. This favorable balance of performance and simplicity positions STOC-T as a promising platform for clinical translation, particularly in functional retinal imaging and high-resolution corneal assessment.

Importantly, the STOC-T architecture is inherently wavelength-independent, as it relies on spatiotemporal modulation of the illumination field and interferometric detection rather than band-specific optical effects. Consequently, the approach can be readily translated to other OCT spectral ranges using standard system configurations, with only conventional adjustments (e.g., source, optics, and dispersion compensation) required to match the selected wavelength band. At present, however, the combined availability of tunable laser sources operating across different spectral ranges together with sensitive, high-speed cameras remains limited. Nevertheless, this constraint is primarily technological in nature and is expected to be alleviated as enabling components continue to advance.

STOC-T remains compatible with phase-sensitive extensions of OCT, as it preserves interferometric detection and retains phase information. The method relies on controlled modulation of the spatial phase distribution across successive acquisitions, while the ballistic component remains correlated, enabling coherent reconstruction. For phase-resolved techniques such as Doppler OCT and polarization-sensitive OCT (PS-OCT), maintaining phase stability within individual acquisitions is essential; accordingly, phase-sensitive analyses can be performed either within single realizations or after appropriate phase referencing across datasets. This suggests that STOC-T can be extended to Doppler and polarization-sensitive measurements, although the specific implementation will depend on the acquisition protocol and synchronization strategy. As mentioned in this paper in the Doppler case (called MLDH), the retrieved signal can provide information analogous to that obtained in laser Doppler holography.

From a translational standpoint, STOC-T can be integrated into existing OCT platforms with minimal hardware overhead, as it relies primarily on standard interferometric components augmented by controlled phase modulation. With automated optimization-based reconstruction, the method has the potential to be deployed in clinical settings without significantly increasing system complexity or operator burden.

Notwithstanding its strengths, current limitations include the cost and bandwidth demands of high-speed cameras, and substantial computational load. Anticipated advances in camera technology, cost reduction, and wider tuning range lasers are expected to further extend imaging depth, resolution, and clinical applicability. Another practical challenge is data volume: a single high-resolution acquisition comprising 32 volumes [see Figs. 10(a)–10(e)] can exceed 8.5 GB, necessitating intelligent strategies for data selection and analysis to constrain storage and processing demands.

A key fundamental limitation remains the dynamic range and sensitivity of STOC-T. Nevertheless, as previously demonstrated,^60^ shot-noise-limited performance (∼80  dB at 5 mW illumination with a 1.7 mm field diameter) is attainable with appropriate measures, including spatial filtering to remove the constant component, albeit with loss of optical carrier information. Importantly, the dynamic range constraint imposed by the concentration of the OC signal in the low-frequency part of the spatial spectrum can be mitigated through multiple acquisitions and postprocessing averaging, which also helps suppress motion artifacts induced by involuntary sample motion (e.g., ocular or tissue movements) during scanning.

Many aspects of the STOC concept remain to be explored. In particular, control over the coherence matrix itself offers potential for further enhancement. In systems that permit precise phase modulation, one could numerically tailor the coherence matrix to recover undisturbed image data. By optimizing orthogonalization of phase masks—analogous to Hadamard coding strategies^38^—it may be possible to maximize acquisition efficiency for a given scatterer’s structure and optical properties. Adaptive selection of phase masks during reconstruction also represents a promising direction.

In summary, STOC-T is a robust, high-performance extension of partial-coherence optical imaging, uniting rapid, scan-free volumetric acquisition with effective speckle suppression and high spatial resolution. Its ability to resolve fine retinal and choroidal structures *in vivo* opens new opportunities for diagnostic imaging in ophthalmology and, more broadly, for high-fidelity imaging in complex scattering media.

## Data Availability

Raw images and data are publicly available in accordance with the guidelines of the original publications.
